# Diversity of Ascomycota in Jilin: Introducing Novel Woody Litter Taxa in *Cucurbitariaceae*

**DOI:** 10.3390/jof8090905

**Published:** 2022-08-26

**Authors:** Wenxin Su, Rong Xu, Chitrabhanu S. Bhunjun, Shangqing Tian, Yueting Dai, Yu Li, Chayanard Phukhamsakda

**Affiliations:** 1 Internationally Cooperative Research Center of China for New Germplasm Breeding of Edible Mushroom, Jilin Agricultural University, Changchun 130118, China; 2 College of Plant Protection, Jilin Agricultural University, Changchun 130118, China; 3 Center of Excellence in Fungal Research, Mae Fah Luang University, Chiang Rai 57100, Thailand; 4 School of Science, Mae Fah Luang University, Chiang Rai 57100, Thailand

**Keywords:** ASAP, fungal barcode, multi-loci phylogeny, northeast China, *Pleosporales*, taxonomy

## Abstract

*Cucurbitariaceae* has a high biodiversity worldwide on various hosts and is distributed in tropical and temperate regions. Woody litters collected in Changchun, Jilin Province, China, revealed a distinct collection of fungi in the family *Cucurbitariaceae* based on morphological and molecular data. Phylogenetic analyses of the concatenated matrix of the internal transcribed spacer (ITS) region, the large subunit (LSU) of ribosomal DNA, the RNA polymerase II subunit (*rpb*2), the translation elongation factor 1-alpha (*tef*1-*α*) and β-tubulin (*β-tub*) genes indicated that the isolates represent *Allocucurbitaria* and *Parafenestella* species based on maximum likelihood (ML), maximum parsimony (MP) and Bayesian analysis (BPP). We report four novel species: *Allocucurbitaria mori*, *Parafenestella changchunensis*, *P. ulmi* and *P. ulmicola*. The importance of five DNA markers for species-level identification in *Cucurbitariaceae* was determined by Assemble Species by Automatic Partitioning (ASAP) analyses. The protein-coding gene *β-tub* is determined to be the best marker for species level identification in *Cucurbitariaceae*.

## 1. Introduction

Fungi are known to have a high diversity; however, the number of named and classified fungi is still lower than the estimated number of species [1,2,3,4]. This could be because several regions are yet to be explored. China is the third largest country in the world by area, with several different climatic conditions [5,6,7,8]. Jilin is a province located in northeast (NE) China where the temperature is hot and dry in summers and has a harsh winter with temperatures down to −20 °C [9]. The vegetation in the eastern mountains includes tree genera such as the *Betula*, *Fraxinus*, *Juglans*, *Larix*, *Pinus*, *Quercus*, *Salix*, *Sorbus* and *Ulmus* [10]. These trees are common in the northern hemisphere and in temperate climates [11].

The family *Cucurbitariaceae* was established by Winter [12], and it is characterized by clustered ascomata and scattered, black, and shiny ostioles, surrounded with olivaceous-to-brown hyphae and having yellow-to-dark olivaceous, brown and muriform ascospores [13,14,15]. Asexual morphs are known to occur as pycnidia with hyaline conidia [14]. *Cucurbitariaceae* has received much attention in recent years, and it includes 13 genera: *Allocucurbitaria* Valenz.-Lopez, Stchigel, Guarro & Cano, *Astragalicola* Jaklitsch & Voglmayr, *Cucitella* Jaklitsch & Voglmayr, *Cucurbitaria* Gray (=*Pleurostromella* Petr.), *Fenestella* Tul. & Tul C., *Neocucurbitaria* Wanas., E.B.G. Jones & K.D. Hyde, *Paracucurbitaria* Valenz.-Lopez Stchigel, Guarro & Cano, *Parafenestella* Jaklitsch & Voglmayr, *Protofenestella* Jaklitsch & Voglmayr, *Rhytidiella* Zalasky, *Seltsamia* Jaklitsch & Voglmayr, *Syncarpella* Theiss. & Syd. and *Synfenestella* Jaklitsch & Voglmayr [13]. Jaklitsch et al. [15] provided a comprehensive study of fenestelloid clades of *Cucurbitariaceae* using fresh collections. Various type specimens were verified, and all the genera of *Cucurbitariaceae* formed a well-supported clade in a multi-locus phylogeny [15]. However, the phylogenetic placement of *Rhytidiella* and *Syncarpella* remain to be confirmed as they lack molecular data [15]. *Fenestella*, *Neocucurbitaria* and *Parafenestella* have a wide distribution mainly in temperate regions and can be found on various hosts [14,16,17,18,19]. For example, *Parafenestella salicum* was found on the twigs of *Salix alba* and *Fenestella parafenestrata* on the branches of *Quercus robur* in Austria, while *Neocucurbitaria subcaespitosa* was isolated from the twigs of *Sorbus aria* in Switzerland [14,15].

This study mainly focuses on ascomycetous fungi from the northern part of China. The novel taxa are introduced based on morphology and molecular data. In this study, *Allocucurbitaria* was used to demonstrate important characteristics for distinguishing the asexual morph at the generic level. This study also determines the best barcode out of five DNA markers for species delineation in *Cucurbitariaceae* by applying assemble species by automatic partitioning (ASAP) analyses.

## 2. Materials and Methods

### 2.1. Collection and Isolation

Dried branches of *Morus alba*, *Populus* species and *Ulmus pumila* were collected from Jilin Agricultural University in Changchun, Jilin Province, China (longitude: 125.410385; latitude: 43.810433). Specimens were kept in sealed paper bags indicating the location, time and host details. The specimens were processed following Senanayake et al. [20] for isolation. Single-spore isolation was performed using potato dextrose agar (PDA) and incubated at 25 °C in the dark [16]. Germinated ascospores were transferred aseptically to PDA and grown at 25 °C for 2 weeks. Pure cultures were deposited at the Engineering Research Center of the Chinese Ministry of Education for Edible and Medicinal Fungi at the Jilin Agricultural University (CCMJ), Changchun, China, and type specimens were deposited in the Herbarium of Mycology, Jilin Agricultural University (HMJAU). The new taxa were registered with Mycobank [17,18].

### 2.2. Morphological Observation

The specimens were examined using a Zeiss Stemi 2000C stereomicroscope equipped with a Leica DFC450C (Leica, Heidelberg, Germany) digital camera. A thin section of partial ascoma was prepared and placed on glass slides with a drop of sterile water. The structure and size of microcharacters were observed and photographed using a digital Axiocam 506 color camera equipped with Zeiss Image A2 (Zeiss, Oberkochen, Germany). Fructification of asexual morph in the sterile culture was observed after four weeks of incubation in the dark.

### 2.3. DNA Extraction, PCR Amplification and Sequencing

Genomic DNA was extracted using NuClean PlantGen DNA Kit (CWBIO, Taizhou, China) according to the manufacturer’s protocol. The internal transcribed spacer region of ribosomal DNA (ITS) [21], the large subunit (LSU) of ribosomal DNA [22], the RNA polymerase II second-largest subunit (*rpb*2) [23], the translation elongation factor 1-alpha (*tef*1*-α*) and beta-tubulin (*β-tub*) were amplified as described in Table 1. The amplification reactions were performed using 20 μL PCR mixtures containing 9 μL of ddH_2_O, 10 μL of 2× EsTaq MasterMix (Dye), 0.4 μL of DNA template and 2 μL of 2 μmol/μL of each forward and reverse primer. All PCR products were visualized with electrophoresis using a 1% agarose gel. The PCR products were sequenced by Sangon Biotech (Shanghai) Co., Ltd., China.

### 2.4. Phylogenetic Analysis

The sequence data were assembled using Geneious Prime 2021 (Biomatters Ltd., Auckland, New Zealand). The closest matches for the new strains were obtained using BLASTn searches (http://www.blast.ncbi.nlm.nih.gov/, accessed on 17 December 2021), and reference sequence data were downloaded from recent publications [14,15]. The sequences were aligned with MAFFT version 7 (https://mafft.cbrc.jp/alignment/server/, accessed on 8 July 2022) [27], and ambiguous nucleotides were manually adjusted following visual examination in AliView version 1.26 [28]. Leading or trailing gaps exceeding the primer binding site were trimmed from the alignments, and the alignment gaps were treated as missing data. The concatenation of the multilocus data was created using Sequence Matrix version 1.8 [29].

Phylogenetic analyses were conducted using maximum likelihood, maximum parsimony and Bayesian inference methods. Maximum likelihood analysis was performed using RAxML-HPC2 on XSEDE on the CIPRES web portal (http://www.phylo.org/portal2/, accessed on 8 July 2022) [30,31,32]. The GTR+I+G model of nucleotide evolution was used for the datasets, and RAxML rapid bootstrapping of 1000 pseudo-replicates was performed [33]. The best-fit evolutionary models for individual and combined datasets were estimated under the Akaike information criterion (AIC) using jModeltest 2.1.10 on the CIPRES web portal for posterior probability [34]. The GTR+I+G model was the best model for the datasets. Maximum parsimony analysis of the combined matrices was performed using a parsimony ratchet approach. Descriptive tree statistics for parsimony (Consistency Index [CI], Homoplasy Index [HI] Tree Length [TL], Retention Index [RI] and Relative Consistency Index [RC]) were calculated for the trees generated under the different optimality criteria. The resulting best trees were then analyzed using PAUP and subjected to a heuristic search with TBR branch swapping (MulTrees option in effect, steepest descent option not in effect) [35]. Bayesian inference analyses were conducted using MrBayes v. 3.2.6 on the CIPRES web portal. Simultaneous Markov chains were run for seven million generations, and trees were sampled every 100th generation [36]. The phylogenetic trees were visualized in FigTree 1.4.3 [37] and edited in Adobe Illustrator CS v. 6 (Adobe, San Jose, CA, USA).

### 2.5. Analysis of Matrix Partitions by Assemble Species by Automatic Partitioning

Puillandre et al. [38] introduced the assemble species by automatic partitioning (ASAP) method to build species partitions. The ASAP method circumscribes species partitions using an implementation of a hierarchal clustering algorithm based on pairwise genetic distances (Kimura 2-Parameter). The pairwise genetic distances are used to build a list of partitions ranked by a score that is computed using the probabilities of groups to define panmictic species. The ASAP delimitations were run on the online version (https://bioinfo.mnhn.fr/abi/public/asap/ (accessed on 13 January 2022)) using single-locus datasets that included 107 strains of *Cucurbitariaceae*. The partition with the lowest ASAP score is known to represent the best partitions [38,39], and thus partitions with the lowest ASAP score were considered for each dataset [39,40].

## 3. Results

### 3.1. Phylogenetic Analyses

The final concatenated dataset comprised 110 ingroup taxa and two outgroup taxa, with 4607 characters including gaps (651 bases for ITS, 911 bases for LSU, 1063 bases for *rpb2*, 1281 bases for *tef*1*-α*, and 701 bases for *β-tub*). The RAxML analysis yielded a best-scoring tree with a final ML optimization likelihood value of −39123.587750. The matrix consisted of 1740 distinct alignment patterns, with 25.90% undetermined characters or gaps. Estimated base frequencies were as follows: A = 0.234707, C = 0.269983, G = 0.265086, T = 0.230223; substitution rates AC = 1.287870, AG = 4.563896, AT = 1.434736, CG = 1.144629, CT = 6.919700, GT = 1.000000; proportion of invariable sites I = 0.606319; gamma distribution shape parameter *α* = 0.967784. The maximum parsimony dataset consisted of 1230 parsimony-informative characters and 246 variable characters. The parsimony analysis yielded 256 most parsimonious trees out of 1000 (TL = 6467, CI = 0.368, RI = 0.806, RC = 0.296, HI = 0.632). In the BPP analysis, 2437 trees were sampled after the 20% burn-in with a stop value of 0.009904. The maximum parsimony dataset consisted of 3132 parsimony-informative characters and 241 variable characters. The parsimony analysis yielded 512 most parsimonious trees out of 1000 (TL = 6468, CI = 0.368, RI = 0.806, RC = 0.297, HI = 0.632). In the BPP analysis, 1461 trees were sampled after the 20% burn-in with a stop value of 0.009955. The phylogenetic trees generated from the ML, MP and BPP had similar topologies (Figures S6 and S7).

**Figure 1:**
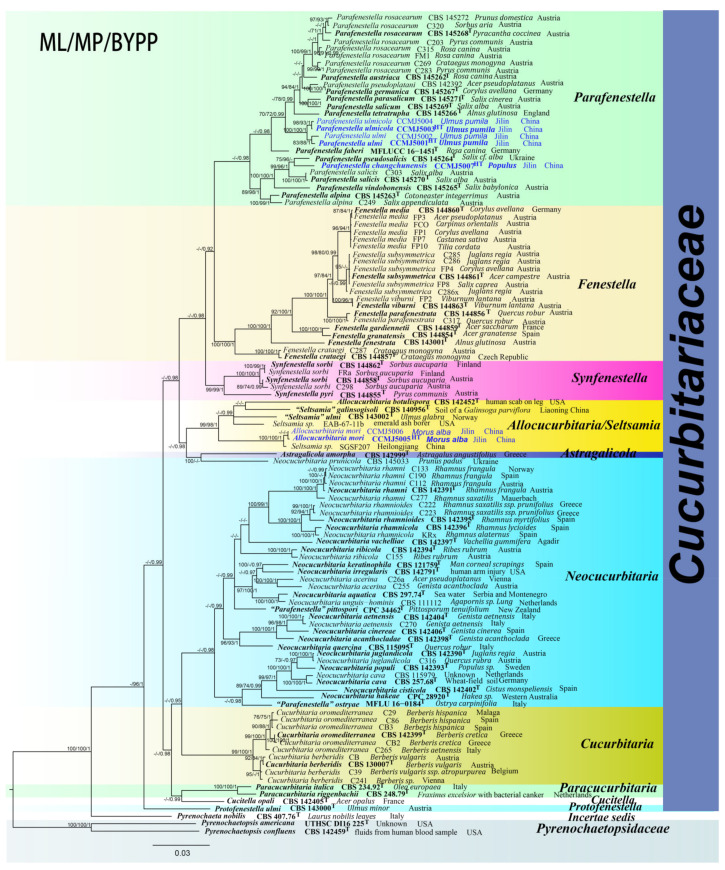
The Bayesian 50% majority-rule consensus phylogram based on a concatenated ITS, LSU, *rpb*2, *tef*1*-α* and *β-tub* dataset of *Cucurbitariaceae*. The tree is rooted with *Pyrenochaetopsis americana* (UTHSC DI16225) and P. confluens (CBS 142459). Bootstrap support values for maximum likelihood and maximum parsimony analysis greater than 70% (ML = left; MP = middle) and Bayesian posterior probabilities ≥ 0.90 (BPP, right) are shown at the nodes. The new species are indicated in blue. The type-derived strains are indicated in bold and marked with ^T^.

In the ML analysis of the ITS region, *Parafenestella ulmi* (CCMJ 5001 and CCMJ 5002) and *P. ulmicola* (CCMJ 5003 and CCMJ 5004) clustered together with high support (ML = 95%), while *P. changchunensis* (CCMJ 5007) formed a clade with *P. vindobonensis* (CBS 145256) with relatively low support (ML = 63%) in *Parafenestella* (Figure S1). *Parafenestella ostryae* (MFLU 16-0184) and *P. pittospori* (CPC 34462) resided in the *Neocucurbitaria* clade (Figure 1) similar to the combined dataset. *Allocucurbitaria mori* (CCMJ 5005 and CCMJ 5006) formed a clade with *A. botulispora* (CBS 142452), *Seltsamia galinsogisoli* (CBS 140956), *S. ulmi* (CBS 143002) and two unidentified *Seltsamia* species (EAB-67-11b and SGSF207) (ML = 100%). The LSU locus could not accurately distinguish taxa at the genus and species level in *Cucurbitariaceae* (Figure S2). In the ML analysis of *β-tub* gene, *P. ulmi* (CCMJ 5001 and CCMJ 5002) and *P. ulmicola* (CCMJ 5003 and CCMJ 5004) formed a clade with high support (ML = 94%), while *P. changchunensis* (CCMJ 5007) clustered with *P. pseudosalicis* (CBS 145264) with moderate support (ML = 71%). *Allocucurbitaria mori* (CCMJ 5005 and CCMJ 5006) and *A. botulispora* (CBS 142452) formed a clade with moderate support (ML = 54%, Figure S5). In the *tef*1-*α* analysis, *Parafenestella ulmi* (CCMJ 5001 and CCMJ 5002) and *P. ulmicola* (CCMJ 5003 and CCMJ 5004) formed a clade with relatively high support (ML = 89%) (Figure S4). *Parafenestella changchunensis* (HMJAU 60182) formed a clade with *P. salicis* (CBS 145270 and C303), *P. pseudosalicis* (CBS 145264), *P. vindobonensis* (CBS 145265) and *P. alpina* (CBS 145263 and C249) with relatively high support (ML = 79%). *Allocucurbitaria mori* (CCMJ 5005 and CCMJ 5006) clustered with *Synfenestella pyri* (CBS 144855) with low support (ML = 41%).

In the multi-locus phylogenetic analysis, *Parafenestella ulmi* (CCMJ 5001 and CCMJ 5002) and *P. ulmicola* (CCMJ 5003 and CCMJ 5004) formed a clade with high support (ML = 100%; MP = 100%; BPP = 1.00). *Parafenestella changchunensis* (CCMJ 5007) clustered with *P. pseudosalicis* (CBS 145264) and *P. salicis (*CBS 145270 and C303*)* with high support (ML = 99%; MP = 96%; BPP = 1.00). *Parafenestella changchunensis* (CCMJ 5007) is closely related to *P. pseudosalicis* (ML = 75%; MP = 96%). The fresh collections from *Morus alba* revealed a new species *Allocucurbitaria mori* (CCMJ 5005 and CCMJ 5006). The two isolates (CCMJ 5005 and CCMJ 5006) formed a close relationship to an unidentified *Seltsamia* species (SGSF207) with strong statistical support (ML = 100%; MP = 100%; BPP = 1.00).

### 3.2. ASAP: Assemble Species by Automatic Partitioning

**Figure 2:**
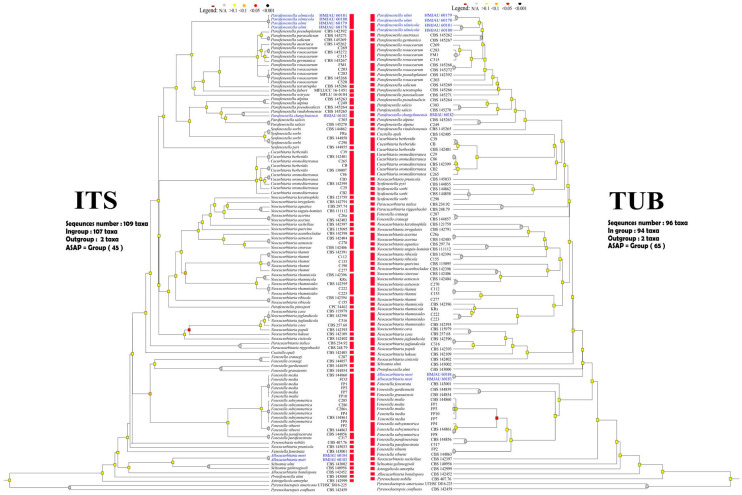
Dendrogram from ASAP analysis based on two datasets (ITS and *β-tub* markers). The results of species delimitation are indicated by red bars. Sequences generated in this study are in blue.

Five single-locus datasets were used that comprised 110 sequences of ITS, 109 sequences of LSU, 101 sequences of *rpb2*, 96 sequences of *β-tub* and 88 sequences of *tef*1*-α*. The ASAP analysis of the ITS region assigned all members of *Cucurbitariaceae* into 45 groups (Figure 2); *β-tub* gene into 65 groups (Figure 2); LSU into 43 groups (Figure S8); *rpb2* gene into 65 groups (Figure S9); *tef*1*-α* gene into 45 groups (Figure S10).

The ASAP analysis recovered *P. ulmi* (CCMJ 5001 and CCMJ 5002), *P. ulmicola* (CCMJ 5003 and CCMJ 5004) and twelve other strains including *P. pseudoplatani* (CBS 142392), *P. austriaca* (CBS 145262), *P. rosacearum* (C203, FM1, C269, C283, CBS 145268, C315, CBS145272, C320), *P. germanica* (CBS 145267) and *P. tetratrupha* (CBS 145266) as one group in the LSU data. *Parafenestella changchunensis* (CCMJ 5007) and *P. pseudosalicis* (CBS 145264) were recovered as one group in the LSU data. The ASAP analysis of the ITS region recovered *P. ulmi* and *P. ulmicola* as one group (Figure 2). The ASAP result of the *β-tub* gene was similar to the combined dataset (Figure 2). *Parafenestella ulmi* and *P. ulmicola* were not delineated by the *tef*1*-α* and *rpb2* genes (Figures S9 and S10). *Parafenestella changchunensis, P. pseudosalicis* (CBS 145264) and *P. salicis* (CBS 145270 and C303) were recovered as one group in the *tef*1*-α* data. *Allocucurbitaria mori* (CCMJ 5005 and CCMJ 5006) grouped with *Synfenestella pyri* (CBS 144855) in the ASAP analysis of the *tef*1*-α* gene, but both were recovered as individual groups in the ITS, LSU, *rpb2*, and *β-tub* datasets.

In the ASAP analysis, the *β-tub* gene was the best marker for identifying *Parafenestella* and *Allocucurbitaria* taxa. *Parafenestella ulmi* and *P. ulmicola* were recovered as a group in ASAP analysis of the ITS and other markers but were recovered as separate groups in the *β-tub* dataset (similar to the combined dataset). *Parafenestella changchunensis* and *P. vindobonensis* (CBS 145265) were recovered as a group in the ITS region but were recovered as distinct species in the *β-tub* dataset. *Allocucurbitaria mori* was recovered as an individual group in all single-marker analyses (except *tef*1*-α* gene). Based on the current results, the *β-tub* gene is the best marker for the identification of *Cucurbitariaceae* taxa at the species level.

### 3.3. Taxonomy

**Figure 3:**
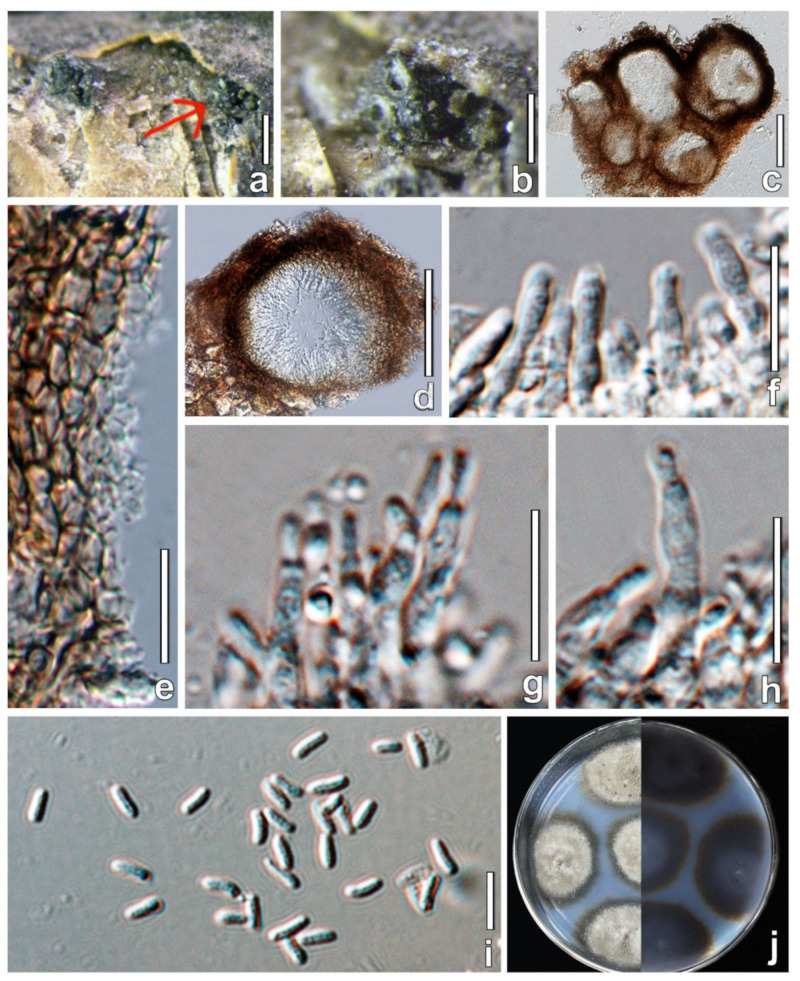
*Allocucurbitaria mori* (HMJAU 60183, holotype) The red arrow indicates the conidiomata in face view. (**a**,**b**) Appearance of conidiomata on host substrate. (**c**,**d**) Vertical section of partial conidiomata. (**e**) Section of partial conidioma wall. (**f**–**h**) Conidiogenous cells and conidia. (**i**) Conidia. (**j**) Culture characteristics on PDA. Scale bars: (**a**) = 500 µm; (**b**) = 200 µm; (**c**,**f**) = 100 µm; (**d**) = 50; (**e**,**g**,**h**) = 10 µm; (**i**) = 5 µm.

***Allocucurbitaria mori*** W.X. Su, Phukhams. & Y. Li, *sp*. *nov*. (Figure 3).

**MycoBank Number**: MB844413.

**Etymology**: Named after the host genus *Morus.*

**Holotype**: HMJAU 60183.

**Description**: *Saprobic* on dead twigs of *Morus alba*. 

**Sexual morph**: Undetermined.

**Asexual morph**: *Stromata* poorly developed, multiloculate, with 5–8 locules forming groups in stromata, immersed. *Conidiomata* 108–180 × 103–201 μm ($\bar{x}$ = 142 × 143 μm, *n* = 6), pycnidia, solitary or aggregated, sometimes confluent, semi-immerged, visible as black protrusions, globose to ellipsoid, coriaceous, black, without distinguishable ostioles. *Pycnidial* wall 5–9 μm wide, thick-walled, composed of 7–10 layers of thin-walled cells of *textura angularis*, dark brown on the outside to gradually lighter on the inside, inner layer subhyaline, lining layer bearing conidiogenous cells. *Conidiophores* reduced to conidiogenous cells. *Conidiogenous cells* 6–14 × 1–5 μm ($\bar{x}$ = 10 × 2 μm, *n* = 30), enteroblastic, solitary, long cylindrical, arising from the inner layer of conidioma, smooth-walled, hyaline. *Conidia* 3–5 × 1–2 μm ($\bar{x}$ = 4 × 1.5 μm, *n* = 50), oblong, hyaline, aseptate, with a minute guttule, smooth.

**Cultural characters**: Colonies on MEA reaching 32–38 mm diam after 4 weeks at 25 °C. Cultures from above, gray at the center, dense in the middle, sparse at the edge, circular, papillate, black lumps produced on the surface of cultures, white at the edge.

**Material examined**: CHINA, Jilin Province, Changchun, Jilin Agricultural University, from *Morus alba* (*Moraceae*) twigs, 20 May 2021, Wenxin Su and C. Phukhamsakda, S057 (HMJAU 60183, **holotype**); ex-type living culture, CCMJ5005; isotype = HMJAU 60184; ex-isotype living culture, CCMJ5006.

**GenBank accession numbers**: CCMJ5005: LSU = OL897171, ITS = OL996120, *tef*1*-α* = OL944601, *rpb2* = OL944505, and *β-tub* = OL898725. CCMJ5006: LSU = OL897172, ITS = OL996121, *tef*1-*α* = OL944602, *rpb*2 = OL944506 and *β-tub* = OL898720.

**Figure 4:**
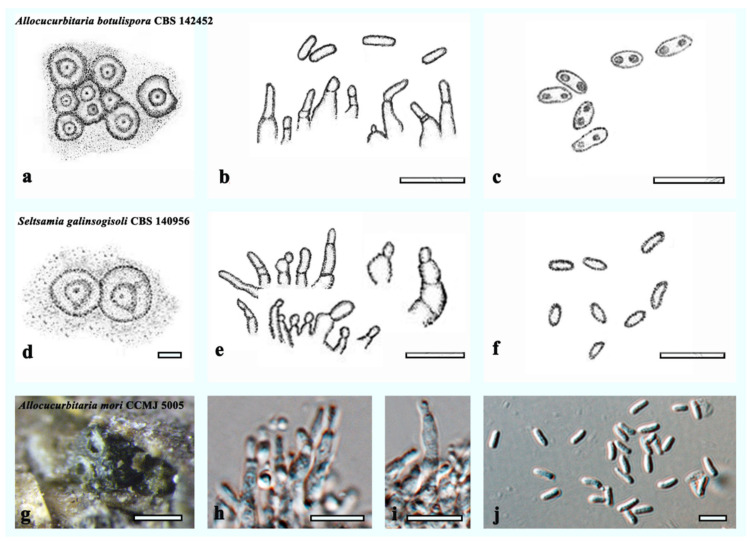
Morphology of related taxa in the *Allocucurbitaria* clade. (**a**–**c**) Characters of *Allocucurbitaria botulispora* were redrawn from Valenzuela-Lopez et al. [41]: (**a**) Pycnidia. (**b**,**c**) Conidiogenous cells and conidia. (**d**–**f**) Characters of *Seltsamia* *galinsogisoli* redrawn from Zhang et al. [42]: (**d**) Pycnidia. (**e**,**f**) Conidiogenous cells and conidia. (**g**–**j**) Characters of *Allocucurbitaria mori* (CCMJ 5005): (**g**) Appearance of conidiomata of *Allocucurbitaria mori* on host substrate. (**h**–**j**) Conidiogenous cells and conidia. Scale bars: (**b**,**c**,**e**,**f**,**h**,**i**) = 10 µm; (**d**) = 20 µm; (**g**) = 200 µm; (**j**) = 5 µm.

**Notes**: *Allocucurbitaria mori* (CCMJ5005 and CCMJ5006) formed a separate clade in *Allocucurbitaria/Seltsamia* with high support (ML = 98%; MP = 97%; BPP = 1.00). Morphologically, *A. mori* (HMJAU 60183) is similar to *A. botulispora* (CBS 142452) and *S. galinsogisoli* (CBS 140956) in having cylindrical, enteroblastic, solitary conidiogenous cells and aseptate conidia [41,42] (Figure 4). However, *S. galinsogisoli* (CBS 140956) has longer conidia, while *A. botulispora* (CBS 142452) has distinct guttulate at the conidia ends [41,42].

A BLASTn search of the ITS region of *A. mori* strain CCMJ 5005 showed a high query cover and similarity (99.80%) to an unidentified *Seltsamia* sp. (SGSF207) from soil. However, there are no other loci available in public databases for comparison. Hence, we introduce *Allocucurbitaria mori* as a novel species, and this is the first report of *Allocucurbitaria* on *Morus* tree [41,42,43].

**Figure 5:**
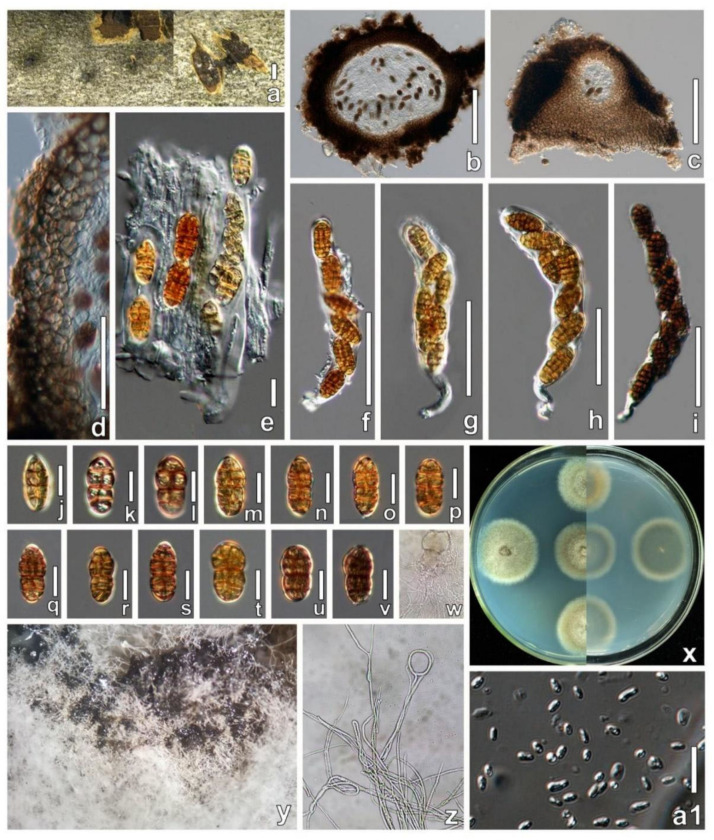
*Parafenestella changchunensis* (HMJAU 60182, holotype). (**a**) Ascomata on host surface. (**b**) Vertical section through partial ascoma. (**c**) Ostioles. (**d**) Partial peridium. (**e**) Pseudoparaphyses. (**f**–**i**) Asci. (**j**–**v**) Development stages of ascospores. (**w**) Germinating ascospore (**x**) Culture characteristics on PDA. (**y**) Pycnidia. (**z**) Hyphal coil structures formed by mycelia. (**a1**) Conidia. Scale bars: (**a**) = 500 µm; (**b**,**c**) = 100 µm; (**d**,**e**) = 20 µm; (**f**–**i**) = 50 µm; (**j**–**v**,**a1**) = 10 µm.

***Parafenestella changchunensis*** W. X. Su, Phukhams. & Y. Li, *sp. nov*. (Figure 5).

**MycoBank Number**: MB844412.

**Etymology**: referring to Changchun City where the sample was collected.

**Holotype**: HMJAU 60182.

**Description**: *Saprobic* on dead stems of *Populus* L.

**Sexual morph**: *Ascomata* 174–416 × 226–486 μm ($\bar{x}$ = 280 × 353 μm, *n* = 5), single or gregarious, scattered, globose to depressed globose, submerged, visible as black dots and protruding host surface, solitary or aggregated. *Ostioles* 61 × 100 μm, center, protruding filled with periphyses. *Peridium* 12–27 μm wide, thick-walled, composed of 6–10 wall layers, outer part comprising dark brown cells of *textura angularis*, inner layer thin-walled, dark brown from the outside radiating light brown cells to hyaline towards the inside. *Hamathecium* of dense, 1.6–2.0 μm ($\bar{x}$ = 1.7 μm, *n* = 10) wide, filamentous, septate, cellular pseudoparaphyses surrounding asci. *Asci* 95–138 × 16–21 μm ($\bar{x}$ = 121 × 18 μm, *n* = 10), 6–8 ascospores, bitunicate, fissitunicate, broad cylindrical, some curved, short-pedicellate, apically rounded with an ocular chamber. *Ascospores* 18–25 × 8–13 μm ($\bar{x}$ = 21 × 10 μm, *n* = 30), uniseriate, partially overlapping, fusiform to oval, slightly asymmetrical, the middle of ascospores is slightly contracted, with 4–6 transverse septa, 2–3 vertical septa, the upper part is slightly larger than the lower part, light yellow to dark brown.

**Asexual morph:** *Pycnidia* produced in PDA after 2 weeks of incubation in the dark, mycelium white. *Conidiomata* confluent or scattered, superficial, covered with dense vegetative hyphae, with turbid whitish drops, globose, black. *Conidia* 5–8 × 2.5–4.5 μm ($\bar{x}$ = 6.5 × 3.7 μm, *n* = 30), oblong to allantoid, hyaline, aseptate, with 1–2 guttules.

**Culture characteristics**: Colonies on PDA, reaching 26–31 mm diam after 2 weeks at 25 °C. Culture from above, mycelium dense and producing hyphal coil structures; from the center to the outer edge, the color changes from grey to greyish-green to white, with obvious concentric wheel patterns, a clear radiation pattern at the back, round.

**Material examined**: CHINA, Jilin Province, Changchun, Jilin Agricultural University, from dead stems of *Populus* L. (*Salicaceae*), 18 April 2021, Wenxin Su, S12-16 (HMJAU 60182, **holotype**); ex-type living culture, CCMJ5007.

**GenBank accession numbers**: CCMJ5007: LSU = OL897170, SSU = OL891808, ITS = OL996119, *tef*1*-α* = OL944600, and *β-tub* = OL898719.

**Notes**: In our phylogenetic analysis, *P. changchunensis* (CCMJ5007) is closely related to *P. pseudosalicis* (CBS 145264) with moderate support (ML = 75%; MP = 96 %; Figure 1). *Parafenestella changchunensis* is morphologically similar to *P. pseudosalicis* in having immersed, concave apex ascomata, with the upper part of young ascospores often wider, ends concolorous and smooth walled [14]. The immature spores of *P. changchunensis* have four horizontal septa and form 2–3 vertical septa during the maturation process. However, the immature spores of *P. pseudosalicis* have 2 transverse septa turning into 2–4 longitudinal septa during the maturation process [15]. *Parafenestella changchunensis* mycelium nodules gradually form fruiting bodies on the medium, while there are no reports of the asexual morph of *P. pseudosalicis* [15].

A BLASTn search of the ITS region of *P. changchunensis* (CCMJ 5007) showed a high similarity and query cover (98.81%) to *P. vindobonensis* (CBS 145265). The *β-tub* sequence of *P. changchunensis* (CCMJ 5007) showed a high query cover and similarity (96.82%) to *P. pseudosalicis* (C301). There were 0.96% (6/627 bases), 0.34% (3/885), 1.78% (13/730) and 7.99% (43/538 bases) base differences in the ITS, LSU, *tef*1-*α* and *β-tub* genes between *P. changchunensis* (CCMJ 5007) and *P. vindobonensis* (CBS 145265), excluding gaps. There were 1.75% (11/627 bases), 0.11% (1/885), 1.10% (8/730) and 3.16% (17/538 bases) base differences in the ITS, LSU, *tef*1-*α* and *β-tub* genes of *P. changchunensis* (CCMJ 5007) and *P. pseudosalicis* strain C301, excluding gaps. Therefore, we introduce *P. changchunensis* as a novel species, and this is the first report of *Parafenestella* on the *Populus* tree [14,15].

**Figure 6:**
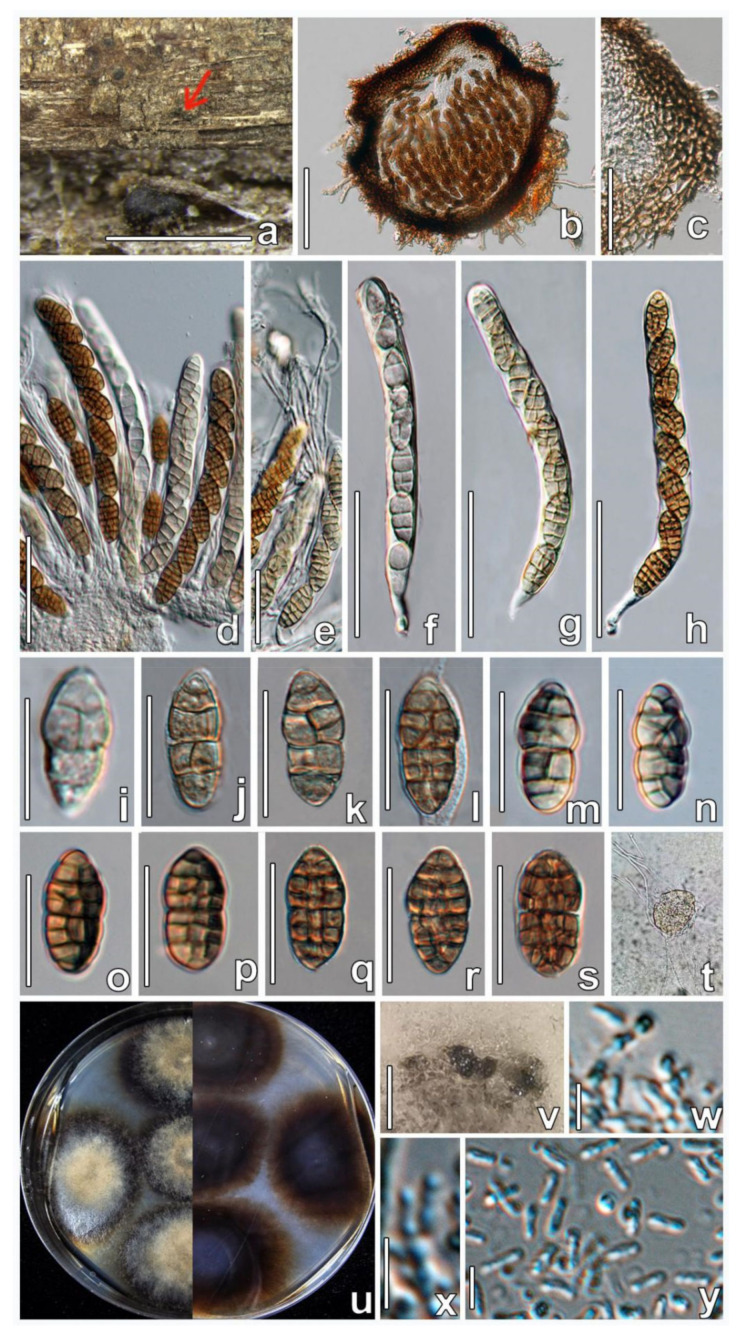
*Parafenestella ulmi* (HMJAU 60178, **holotype**). (**a**) Ascomata on host surface. (**b**) Vertical section through ascoma. (**c**) Partial peridium in vertical section. (**d**,**e**) Asci arrangement along with pseudoparaphyses. (**f**–**h**) Development stages of asci. (**i**–**s**) Development stages of ascospores. (**t**) Germinating ascospore. (**u**) Four-week-old culture characteristics on PDA. (**v**) Pycnidia formed in sterile culture after two weeks of incubation on PDA. (**w**,**x**) Conidiogenous cells and conidia. (**y**) Conidia. Scale bars: (**a**) = 500 µm; (**b**) = 100 µm; (**c**–**h**) = 50 µm; (**i**–**s**) = 20 µm; (**v**) = 200 µm; (**w**–**y**) = 5 µm.

***Parafenestella ulmi*** W.X. Su, Phukhams., & Y. Li, *sp. nov.* (Figure 6).

**MycoBank Number**: MB844410.

**Etymology:** Named after the host genus *Ulmus.*

**Holotype**: HMJAU 60178.

**Description**: *Saprobic* on dead stems of *Ulmus pumila*.

**Sexual morph**: *Ascomata* 170–225 × 194–260 μm ($\bar{x}$ = 201 × 229 μm, *n* = 5), immersed, visible as black spots or having a convex surface, solitary, scattered, globose to ellipsoid, flat at the base, coriaceous, black. *Peridium* 19–39 μm wide, composed of 6–10 layers, outer part comprising dark brown cells of *textura angularis*, inner layer comprising thin-walled, light brown cells of *textura angularis*. *Hamathecium* of dense, 1.5–4.5 μm wide ($\bar{x}$ = 2.2 μm, *n* = 20), filamentous, septate, pseudoparaphyses surrounding asci. *Asci* 115–181 × 11–15 μm ($\bar{x}$ = 132 × 13 μm, *n* = 20), 8 ascospores, bitunicate, cylindrical, mostly curved, short-pedicellate, apically rounded with an ocular chamber, clearly visible when immature. *Ascospores* 18–24 × 8–12 μm ($\bar{x}$ = 22 × 10 μm, *n* = 30), uniseriate to partially overlapping, broadly ellipsoid, slightly pointed at both ends, 5–8 transversely septate, 1–2 vertically septate, mature spores constricted at the middle septum, slightly curved, initially hyaline, becoming yellowish to brown at maturity, the cell above median septum slightly wider, smooth-walled.

**Asexual morph**: *Pycnidia* produced in PDA after 2 weeks of incubation in the dark, mycelium greenish, 1–3 μm ($\bar{x}$ = 2.2 μm, *n* = 20), uniloculate, confluent or scattered, superficial, covered with dense vegetative hyphae, globose, dark brown to black. *Conidiogenous cells* 18–24 × 8–12 μm ($\bar{x}$ = 22 × 10 μm, *n* = 30), enteroblastic, phialidic, determinate, discrete, solitary, short cylindrical or conical, straight, with broad base, hyaline. *Conidia* 3–5 × 1–2 μm ($\bar{x}$ = 4.3 × 1.5 μm, *n* = 30), long ellipsoid to cylindrical, aseptate, with two small guttulate at the polar ends, hyaline, smooth-walled.

**Culture characteristics**: Colonies on PDA, reaching 45–48 mm diam after two weeks at 25 °C. Culture from above the center to the outer edge, the color radiating from black to dark green to yellow and white edges, with obvious concentric wheel patterns, dense intermediate hyphae and sparse white mycelium at the outer circle; reverse greenish-black, round.

**Material examined**: CHINA, Jilin Province, Changchun, Jilin Agricultural University, from *Ulmus pumila* (*Ulmaceae*) stem litter, 15 March 2021, Wenxin Su and C. Phukhamsakda, S12 (HMJAU 60178, **holotype**); ex-type living culture, CCMJ 5001, isotype = HMJAU 60179; ex-isotype living culture, CCMJ 5002.

**GenBank accession numbers**: CCMJ5001: LSU = OL897166, SSU = OL891806, ITS = OL996115, *tef*1-*α* = OL944596, *rpb2* = OL944501, and *β-tub* = OL898723. CCMJ5002: LSU = OL897167, ITS = OL996116, *tef*1-*α* = OL944597, *rpb*2 = OL944502, and *β-tub* = OL898717.

**Notes**: In our phylogenetic analysis, *P. ulmi* (CCMJ 5001 and CCMJ 5002) and *P. ulmicola* (CCMJ 5003 and CCMJ 5004) formed a clade in *Parafenestella* with high statistical support (ML = 100%; MP = 100%; BPP = 1.00; Figure 1). Both *P. ulmi* and *P. ulmicola* were found on dead branches of *Ulmus pumila* in Jilin Province, China, which lies in the temperate zone. *Parafenestella* taxa are mainly recorded in Austria, followed by England, Germany and Ukraine, which are all temperate countries [15]. Morphologically, the ascomata of *P. ulmi* and *P. ulmicola* are semi-immersed, visible as black spots or convex surfaces. The asci of *P. ulmi* are longer than *P. ulmicola* but similar in width (132 × 13 vs. 119 × 13 μm). The immature ascospores of *P. ulmi* present 2–3 transverse septa without longitudinal septate, but the spores have 4–8 transverse septa with 1–3 longitudinal septate at mature stages. The ascospores of *P. ulmicola* showed indentation when immature that disappeared during maturation. The ascospores of *P. ulmicola* showed 5–8 transverse septa and 1–2 vertically septate after maturity with less constriction at the septum. The ascospores of *P. ulmi* are yellowish to brown, while *P. ulmicola* have dark brown ascospores at maturity. In PDA, the colonies of *P. ulmicola* have wavy and aggregated colony edges. The colonies of *P. ulmi* are blue-black (reverse view) with black-green edges, while *P. ulmicola* is gray-brown with white edges.

A BLASTn search of the ITS region of *P. ulmi* strain CCMJ 5001 showed a high query cover and similarity (96.45%) to *P. tetratrupha* (CBS 145266) while the *β-tub* sequence of *P. ulmi* strain CCMJ 5001 showed a high similarity and query cover (97.07%) to *P. germanica* strain C307. Therefore, we introduce *P. ulmi* as a novel species.

**Figure 7:**
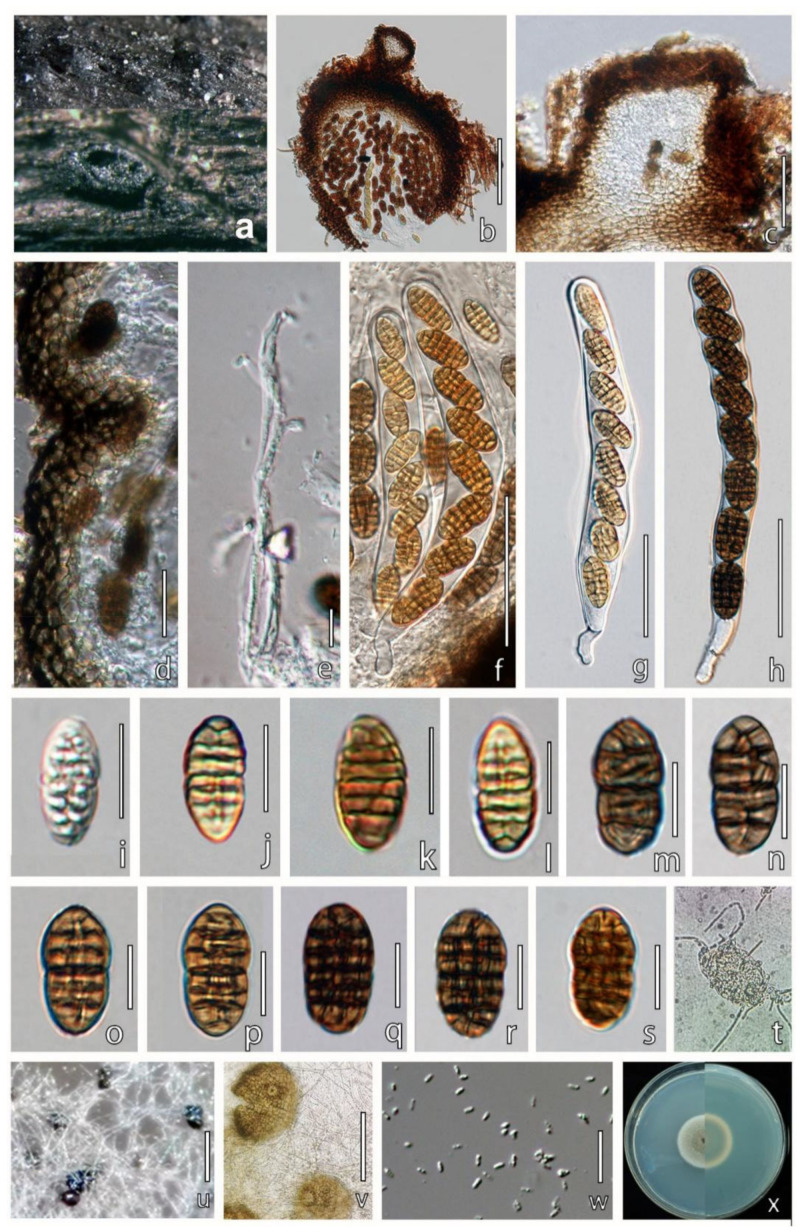
*Parafenestella ulmicola* (HMJAU 60180, holotype). (**a**) Ascomata on host surface. (**b**) Vertical section through ascoma. (**c**) Ostiole. (**d**) Partial peridium wall. (**e**) Pseudoparaphyses. (**f**–**h**) Asci. (**i**–**s**) Developmental stages of ascospores. (**t**) Germinating ascospore. (**u**) Pycnidia produced in four weeks old cultures on PDA. (**v**) Conidiomata. (**w**) Conidia. (**x**) Four weeks old culture on PDA. Scale bars: (**b**) = 100 µm; (**c**) = 50 µm; (**d**,**e**) = 20 µm; (**f**–**h**) = 50 µm; (**i**–**s**) = 10 µm; (**u**) = 200 µm; (**v**) = 100 µm; (**w**) = 5 µm.

***Parafenestella ulmicola*** W.X. Su, Phukhams., & Y. Li, *sp*. *nov*. (Figure 7).

**MycoBank Number**: MB844411.

**Etymology**: Named after the host genus *Ulmus*.

**Holotype**: HMJAU 60180.

**Description**: *Saprobic* on twigs debris of *Ulmus pumila* L. 

**Sexual morph**: *Ascomata* 242–434 × 310–462 μm ($\bar{x}$ = 306 × 359 μm, *n* = 5) μm wide, semi-immersed, visible as a convex hemisphere, globose to subglobose, solitary or mostly aggregated, scattered, coarse-walled, coriaceous, black, with a papilla. *Ostiole* 21 × 24 μm, centrally located. *Peridium* 21–68 μm wide, composed of 11–20 wall layers, with dark brown cells of *textura angularis*. *Asci* 105–153 × 11–14 μm ($\bar{x}$ = 119 × 13 μm, *n* = 20), 8 ascospores, bitunicate, fissitunicate, broadly cylindrical, apically rounded, some curved, short-pedicellate, ocular chamber is not visible at maturity. *Ascospores* 17–22 × 8–12 μm ($\bar{x}$ = 19 × 9 μm, *n* = 30), uniseriate, rarely overlapping, broadly oval, blunt at both ends, narrow towards the ends, with 4–8 transversely septate, 1–3 vertically septate, constricted at the middle septum, initially hyaline, becoming yellowish to brown at maturity, smooth-walled. 

**Asexual morph**: *Pycnidia* produced in cultures on PDA after four weeks of incubation in the dark, mycelium greenish, 41–158 μm diam, covered with white mycelium, ellipsoid, semi-immersed, scattered or aggregated, black, ostiole central. *Peridium* with brown cells of *textura angularis*. *Conidia* 1.4–2.5 × 0.6–0.9 μm ($\bar{x}$ = 1.9 × 0.7 μm, *n* = 30), cylindrical to allantoid, hyaline, smooth, aseptate, with a minute guttulate.

**Culture characteristics**: Colonies on PDA reaching 35–41 mm diam after 2 weeks at 25 °C. Culture from above the center to the outer edge, the color changes from grey to taupe to white, with obvious concentric wheel patterns; a few weeks later, the outer circle hyphae grow into round dark green hyphae with a thin surface.

**Material examined:** CHINA, Jilin Province, Changchun, Jilin Agricultural University, from Ulmus pumila (Ulmaceae) twigs debris, 15 March 2021, Wenxin Su and C. Phukhamsakda, S16 (HMJAU 60180, **holotype**); ex-type living culture, CCMJ 5003, isotype = HMJAU 60181; ex-isotype living culture, CCMJ 5004.

**GenBank accession numbers**: CCMJ5003: LSU = OL897168, SSU = OL891807, ITS = OL946117, *tef*1*-α =* OL944598, *rpb*2 = OL944503 and *β-tub* = OL898724. CCMJ5004: LSU = OL897169, ITS = OL996118, *tef*1-*α* = OL944599, *rpb*2 = OL944504 and *β-tub* = OL898719.

**Notes**: Sixteen *Parafenestella* species are listed in Species Fungorum [44], of which six species were reported on *Rosaceae,* four on *Salicaceae* and three on *Betulaceae*, while one species was reported on *Pittosporaceae, Salicaceae* and *Sapindaceae* [14,15,45,46]. *Parafenestella ulmicola* (CCMJ 5003 and CCMJ 5004) is closely related to *P. ulmi* (CCMJ 5001 and CCMJ 5002) within *Parafenestella* (ML = 100%; MP = 100%; BPP = 1.00, Figure 1). There were 2.31% (12/518) base differences in the *β-tub*, 0.14% (1/733) base differences in the *tef*1-*α* and 0.27% (2/736) base differences in the *rpb*2 gene between *P. ulmicola* (CCMJ 5003 and CCMJ 5004) and *P. ulmi* (CCMJ 5001 and CCMJ 5002), excluding gaps. There were no base differences in the ITS and LSU sequences.

*Parafenestella ulmi* and *P. ulmicola* are phylogenetically close to *P. tetratrupha* but differ from *P. tetratrupha* by having a less longitudinal septa being visible at the surface [20]. *Parafenestella tetratrupha* ascospores are ellipsoid, yellow-brown to reddish-brown to dark brown, with 1–3 main septa, 8–17 distinct transverse and 2–4 longitudinal septa; they are darker and longer than *P. ulmi* and *P. ulmicola* (26.5–33.5 × 13–16.5 vs. 18–24 × 8–12 vs. 17–22 × 8–12 µm) and have more transverse septa than *P. ulmi* and *P. ulmicola* (Table 2). In the multi-locus phylogenetic analysis, although *P. rosacearum* was divided into six groups (Figure 1), it was still identified as one species because the *tef*1-*α* sequences of C203, C283 and C309 are almost the same. The *rpb*2 sequences of strains C203, C315, FM1 and FP11 are identical, while C269 and C283 differ from C203, C283 and C309 by 20 nucleotides [15]. In the phylogenetic analysis, *P. germanica* and *P. pseudoplatani* clustered in the same clade as *P. parasalicum* and *P. salicum*. These strains were identified as different species due to morphological distinctiveness [15]. The ascospores of *P. germanica* were larger than *P. pseudoplatani* (29–39.5 × 13–16.5 vs. 25–29 × 12–14 µm). The ascospores of *P. parasalicum* were larger than *P. salicum* (36–44 × 15.8–19.3 vs. 27–33 × 12.5–16 µm) (Table 3). There were 0.40% (2/494) base differences in the ITS, 2.28% (16/701) base differences in *β-tub*, 0.51% (4/789) base differences in *tef1*-*α* and 1.41% (15/1063) base differences in *rpb*2 between *P. germanica* and *P. pseudoplatani*. There were 3.42% (24/701) base differences in *β-tub*, 1.90% (15/789) base differences in *tef1-α* and 1.32% (14/1063) base differences in *rpb*2 between *P. parasalicum* and *P. salicum.* Thus, the species boundaries of *P.ulmi* and *P. ulmicola* were justified based on their distinct morphological traits and nucleotides differences. Therefore, we introduce *P. ulmicola* as a novel species, and this is first report of *Parafenestella* on *Ulmus* trees.

## 4. Discussion

The family *Cucurbitariaceae* was introduced by Winter [12] and typified by *Cucurbitaria berberidis* (Pers.) Gray [46]. Members of this family occur worldwide and are commonly recorded in Austria, Germany, England and Ukraine as saprobic or necrotrophic on various substrates including plant debris, soil and wood [14,15,47]. Although ribosomal markers and the ITS region are important for phylogenetic analyses, other loci are often needed for better resolution at the species level [48,49,50,51]. The ITS region can have low support values on key evolutionary nodes and cannot be used to accurately classify species in most genera [52,53]. Housekeeping genes and protein-coding genes such as *act*, *β-tub*, *cal*, *gapdh*, *rpb*2 and *tef*1*-α* are thus usually recommended for a stable and reliable topology in phylogenetic analyses [54,55,56].

In this study, ASAP [38] was used to determine the most informative loci for *Parafenestella*. The *β-tub* gene provided the best species level identification of *Parafenestella,* followed by *rpb*2, *tef*1-*α*, ITS and LSU based on ASAP analyses (Figure 2, Figures S8–S11). ASAP analyses based on the *β-tub* gene provided the best resolution of *P. ulmi* and *P. ulmicola*, in addition to *P. changchunensis*, *P. pseudosalicis* and *P. salicis* (Figure 2). The ITS region is an important marker; however, it could not delineate between *P*. *pseudoplatani* (CBS 142392), *P. parasalicum* (CBS 145271), *P. salicum* (CBS 145269), *P. austriaca* (CBS 145262), *P. germanicola* (CBS 145267) and *P. rosacearum* (C203, C269, C283, C315, C320, CBS 145272, CBS 145268, FM1) as they were recovered as a group in ASAP analysis. In the ASAP analysis of the *β-tub* gene, this clade was divided into seven groups: (1) *P. austriaca* (CBS 145262), (2) *P. germanicola* (CBS 145267), (3) *P. rosacearum* (C269, C283, C315, FM1), (4) *P. rosacearum* (CBS 145272, CBS 145268) and *P. rosacearum* (C203), (5) *P. pseudoplatani* (CBS 142392), (6) *P. parasalicum* (CBS 145271) and (7) *P. salicum* (CBS 145269) (Figure 2). The *β-tub* gene exists in all eukaryotes and is involved in the formation of the spindle during cell division [57]. *β*-tubulin plays an important role in defining the characteristics of species [58]. The ASAP analysis of the *β-tub* gene likely reflects the interspecific relationship within *Parafenestella*. Thus, we encourage the inclusion of *β-tub* in the phylogenetic studies of *Parafenestella* species. This result is also supported by the phylogeny of single genes, two loci datasets (ITS + *β-tu*b, Figure S14); ITS + *rpb*2, Figure S12); ITS + *tef*1-*α*, Figure S13) and multi-loci dataset (Figures S7 and S11).

Valenzuela-Lopez et al. [58] established *Allocucurbitaria* in *Cucurbitariaceae* based on morphological and phylogenetic analysis*. Allocucurbitaria botulispora* (CBS 142452) was classified as *Pyrenochaeta* species [43]. Valenzuela-Lopez et al. [41] examined the morphology of *Pyrenochaeta* and suggested that *A. botulispora* was more similar to phoma-like taxa. As it clustered in *Cucurbitariaceae*, the authors classified the species under the genus *Allocucurbitaria* within *Cucurbitariaceae* [41]. *Seltsamia* was introduced with the unique characteristics of pleomassaria-like fungus [14]. There is no confirmed report of the holomorph character of the type species (*S. ulmi*), and thus the generic status is constrained. Three species of *Allocucurbitaria* are listed in Species Fungorum [44], with one species reported on *Ulmus glabra* in Norway*,* one species from soil in China and one species reported from diseased human scab in the USA [41,59]. Notably, the *Allocucurbitaria* strains can be saprophyte and can harbor soil and/or opportunistic fungal disease in humans [41,42,43]. We provide the first report of *Allocucurbitaria* on dead twigs of *Populus morus*.

*Parafenestella* is the fourth most speciose genera in *Cucurbitariaceae* (*Cucurbitaria* 94 species; *Fenestella* 28 species; *Neocucurbitaria* 21 species; *Parafenestella* 14 species; *Syncarpella* 7 species; *Rhytidiella* 4 species; *Allocucurbitaria* 2 species; *Astragalicola* 2 species; *Paracucurbitaria* 2 species; *Synfenestella* 2 species; *Cucitella* 1 species; *Protofenestella* 1 species; *Seltsamia* 1 species) [44]. *Parafenestella* species are commonly distributed over temperate areas including northeast China but are rarely found in the tropical regions [11,13]. All three novel species in this study were collected during early spring in Changchun, Jilin Province, China. Jilin Province (40°52′~46°18′ N) belongs to a temperate continental climate, and the study of similar vegetation from similar climates is likely to result in many *Parafenestella* taxa [60]. We speculate that extensive investigations in the temperate regions would result in numerous *Parafenestella* members. Climate conditions also affect the infection degree of *Cucurbitariaceae* fungi to hosts, as temperatures below 0 °C may stop fungal development [15]. The age of the host including branch size and thickness may also affect the development of *Cucurbitariaceae* [15].

*Parafenestella* is characterized by immersed to erumpent and aggregated or clusters of ascomata [15]. The number of ascomata in *Parafenestella* (as a cluster) is often less than 10, which is higher than in *Fenestella* and *Synfenestella* [14,15]. *Parafenestella* does not form distinct pseudostromata, while *Fenestella* forms a pustular pseudostroma appearing as bumps, and *Synfenestella* forms conspicuous pseudostromatic pustules on pseudostromata [15]. The ascospores of *Parafenestella* are irregularly arranged and partially overlapping, while the ascospores of *Fenestella* and *Synfenestella* are borne in a uniseriate arrangement [14,15]. The sexual morph of *Cucurbitariaceae* is usually found on the wood and bark of trees and shrubs (*Corylus avellana*, *Prunus domestica*, *Rosa canina*, *Sorbus aucuparia*) [15]. The asexual morph of *Parafenestella* has not been reported from the natural host and is successfully produced only in culture [14,15]. However, pycnidia in artificial culture often lack conidiophores, which could be due to environmental conditions [61].

## Figures and Tables

**Table 1:** The PCR primers and amplifying conditions used in this study.

Amplification Loci (Primer Pair Forward/Reverse)	PCR Conditions	References
ITS (ITS5/ITS4)	An initial denaturation step of 5 min at 94 °C, followed by 35 cycles of 30 s at 94 °C, 30 s at 56 °C and 90 s at 72 °C, and a final extension step of 10 min at 72 °C, and 10 °C for holding temperature	White et al. [21]
*rpb*2 (fRPB2-5F/fRPB2-7cR)		Vilgalys et al. [23]
*tef*1*-α* (2218F/983R)		Carbone and Kohn [24] Rehner and Buckley [25]
LSU (LROR/LR5)	An initial denaturation step of 5 min at 94 °C, followed by 35 cycles of 30 s at 94 °C, 45 s at 53 °C and 90 s at 72 °C, and a final extension step of 10 min at 72 °C, and 10 °C for holding temperature	Vilgalys and Hester [22]
*Β-tub* (T1/Bt2b)		O’Donnell and Cigelnik [26]
	

**Table 2:** The dataset used for phylogenetic analysis. The type-derived sequences are in bold.

Taxon	Strain	Host/Substrate	Typification Status	GenBank Accession Numbers				
				ITS	LSU	*rpb2*	*tef*1-*α*	*β-tub*
***Allocucurbitaria botulispora***	**CBS 142452**	**human scab on leg**	**Holotype**	**LT592932**	**LN907416**	**LT593070**	**–**	**LT593001**
***Allocucurbitaria mori***	**HMJAU 60183**	***Morus alba***	**Holotype**	**OL996120**	**OL897171**	**OL944505**	**OL944601**	**OL898725**
***Allocucurbitaria mori***	**HMJAU 60184**	***Morus alba***	**Isotype**	**OL996121**	**OL897172**	**OL944506**	**OL944602**	**OL898720**
***Astragalicola amorpha***	**CBS 142999**	***Astragalus angustifolius***	**Holotype**	**MF795753**	**MF795753**	**MF795795**	**MF795842**	**MF795883**
***Cucitella opali***	**CBS 142405**	***Acer opalus***	**Holotype**	**MF795754**	**MF795754**	**MF795796**	**MF795843**	**MF795884**
*Cucurbitaria berberidis*	C39	*Berberis vulgaris subsp. atropurpurea*	–	MF795755	MF795755	MF795797	MF795844	MF795885
*Cucurbitaria berberidis*	CB	*Berberis vulgaris*	–	MF795757	MF795757	MF795799	MF795846	MF795887
***Cucurbitaria berberidis***	**CBS 130007 = CB1**	***Berberis vulgaris***	**Epitype**	**MF795758**	**MF795758**	**MF795800**	**–**	**–**
*Cucurbitaria berberidis*	CBS 142401 = C241	*Berberis* sp.	–	MF795756	MF795756	MF795798	MF795845	MF795886
*Cucurbitaria oromediterranea*	C265	*Berberis aetnensis*	–	MF795762	MF795762	MF795804	MF795850	MF795891
*Cucurbitaria oromediterranea*	C29	*Berberis hispanica*	–	MF795759	MF795759	MF795801	MF795847	MF795888
*Cucurbitaria oromediterranea*	C86	*Berberis hispanica*	–	MF795760	MF795760	MF795802	MF795848	MF795889
*Cucurbitaria oromediterranea*	CB2	*Berberis cretica*	–	MF795763	MF795763	MF795805	MF795851	MF795892
*Cucurbitaria oromediterranea*	CB3	*Berberis hispanica*	–	MF795764	MF795764	MF795806	MF795852	–
***Cucurbitaria oromediterranea***	**CBS 142399 = C229**	***Berberis cretica***	**Holotype**	**MF795761**	**MF795761**	**MF795803**	**MF795849**	**MF795890**
*Fenestella crataegi*	C287	*Crataegus monogyna*	–	MK356281	MK356281	–	MK357554	MK357598
***Fenestella crataegi***	**CBS 144857 = C314**	***Crataegus monogyna***	**Epitype**	**MK356282**	**MK356282**	**MK357512**	**MK357555**	**MK357599**
***Fenestella fenestrata***	**CBS 143001 = FP9**	***Alnus glutinosa***	**Epitype**	**MF795765**	**MF795765**	**MF795807**	**MF795853**	**MF795893**
***Fenestella gardiennetii***	**CBS 144859 = FM**	***Acer saccharum***	**Holotype**	**MK356283**	**MK356283**	**MK357513**	**MK357556**	**MK357600**
***Fenestella granatensis***	**CBS 144854 = C279**	***Acer granatense***	**Holotype**	**MK356284**	**MK356284**	**MK357514**	**MK357557**	**MK357601**
***Fenestella media***	**CBS 144860 = FP**	***Corylus avellana***	**Epitype**	**MK356285**	**MK356285**	**MK357515**	**MK357558**	**MK357602**
*Fenestella media*	FCO	*Carpinus orientalis*	–	MK356286	MK356286	MK357516	MK357559	–
*Fenestella media*	FP1	*Corylus avellana*	–	MK356287	MK356287	MK357517	MK357560	MK357603
*Fenestella media*	FP3	*Acer pseudoplatanus*	–	MK356288	MK356288	MK357518	MK357561	MK357604
*Fenestella media*	FP7	*Castanea sativa*	–	MK356289	MK356289	MK357519	MK357562	MK357605
*Fenestella media*	FP10	*Tilia cordata*	–	MK356290	MK356290	MK357520	MK357563	MK357606
***Fenestella parafenestrata***	**CBS 144856 = C306**	***Quercus robur***	**Holotype**	**MK356291**	**MK356291**	**MK357521**	**MK357564**	**MK357607**
*Fenestella parafenestrata*	C317	*Salix* sp.	–	MK356292	MK356292	MK357522	MK357565	MK357608
***Fenestella subsymmetrica***	**CBS 144861 = FP6**	***Acer campestre***	**Holotype**	**MK356297**	**MK356297**	**MK357525**	**MK357569**	**MK357610**
*Fenestella subsymmetrica*	C285	*Juglans regia*	–	MK356293	MK356293	MK357523	MK357566	–
*Fenestella subsymmetrica*	C286	*Juglans regia*	–	MK356294	MK356294	–	MK357567	–
*Fenestella subsymmetrica*	C286x	*Juglans regia*	–	MK356295	MK356295	–	–	–
*Fenestella subsymmetrica*	FP4	*Corylus avellana*	–	MK356296	MK356296	MK357524	MK357568	MK357609
*Fenestella subsymmetrica*	FP8	*Salix caprea*	–	MK356298	MK356298	MK357526	MK357570	MK357611
***Fenestella viburni***	**CBS 144863 = FVL**	***Viburnum lantana***	**Holotype**	**MK356300**	**MK356300**	**MK357528**	**MK357572**	**MK357613**
*Fenestella viburni*	FP2	*Viburnum lantana*	–	MK356299	MK356299	MK357527	MK357571	MK357612
***Neocucurbitaria acanthocladae***	**CBS 142398 = C225**	***Genista acanthoclada***	**Holotype**	**MF795766**	**MF795766**	**MF795808**	**MF795854**	**MF795894**
*Neocucurbitaria acerina*	C26a	*Acer pseudoplatanus*	–	MF795767	MF795767	MF795809	MF795855	MF795895
*Neocucurbitaria acerina*	CBS 142403 = C255	*Acer pseudoplatanus*	–	MF795768	MF795768	MF795810	MF795856	MF795896
***Neocucurbitaria aetnensis***	**CBS 142404 = C261**	***Genista aetnensis***	**Holotype**	**MF795769**	**MF795769**	**MF795811**	**MF795857**	**MF795897**
*Neocucurbitaria aetnensis*	C270	*Genista aetnensis*	–	MF795770	MF795770	MF795812	MF795858	MF795898
***Neocucurbitaria aquatica***	**CBS 297.74**	**Sea water**	**Holotype**	**LT623221**	**EU754177**	**LT623278**	**–**	**LT623238**
*Neocucurbitaria cava*	CBS 115979	–	–	AY853248	EU754198	LT623273	–	LT623234
***Neocucurbitaria cava***	**CBS 257.68**	**Wheat-field soil**	**Epitype**	**JF740260**	**EU754199**	**LT717681**	**–**	**KT389844**
***Neocucurbitaria cinereae***	**CBS 142406 = KU9**	***Genista cinerea***	**Holotype**	**MF795771**	**MF795771**	**MF795813**	**MF795859**	**MF795899**
***Neocucurbitaria cisticola***	**CBS 142402 = C244**	***Cistus monspeliensis***	**Holotype**	**MF795772**	**MF795772**	**MF795814**	**MF795860**	**MF795900**
***Neocucurbitaria hakeae***	**CBS 142109 = CPC 28920**	***Hakea*** **sp.**	**Holotype**	**KY173436**	**KY173526**	**KY173593**	**–**	**KY173613**
***Neocucurbitaria irregularis***	**CBS 142791**	**Subcutaneous tissue from injured human arm**	**Holotype**	**LT592916**	**LN907372**	**LT593054**	**–**	**LT592985**
*Neocucurbitaria juglandicola*	C316	*Quercus rubra*	–	MK356301	MK356301	MK357529	MK357573	MK357614
***Neocucurbitaria juglandicola***	**CBS 142390 = BW6**	***Juglans regia***	**Holotype**	**MF795773**	**MF795773**	**MF795815**	**MF795861**	**MF795901**
***Neocucurbitaria keratinophila***	**CBS 121759**	**From human corneal scrapings (keratitis)**	**Holotype**	**EU885415**	**LT623215**	**LT623275**	**–**	**LT623236**
***Neocucurbitaria populi***	**CBS 142393 = C28**	***Populus*** **sp.**	**Holotype**	**MF795774**	**MF795774**	**MF795816**	**MF795862**	**MF795902**
*Neocucurbitaria prunicola*	CBS 145033	*Prunus padus*	–	MK442594	MK442534	MK442668	–	MK442737
***Neocucurbitaria quercina***	**CBS 115095**	***Quercus robur***	**Neotype**	**LT623220**	**GQ387619**	**LT623277**	**–**	**LT623237**
***Neocucurbitaria rhamni***	**CBS 142391 = C1**	***Rhamnus frangula***	**Epitype**	**MF795775**	**MF795775**	**MF795817**	**MF795863**	**–**
*Neocucurbitaria rhamni*	C112	*Rhamnus frangula*	–	MF795776	MF795776	MF795818	MF795864	MF795903
*Neocucurbitaria rhamni*	C133	*Rhamnus frangula*	–	MF795777	MF795777	MF795819	MF795865	MF795904
*Neocucurbitaria rhamni*	C190	*Rhamnus frangula*	–	MF795778	MF795778	MF795820	MF795866	–
*Neocucurbitaria rhamni*	C277	*Rhamnus saxatilis*	–	MF795779	MF795779	MF795821	MF795867	MF795905
***Neocucurbitaria rhamnicola***	**CBS 142396 = C185**	***Rhamnus lycioides***	**Holotype**	**MF795780**	**MF795780**	**MF795822**	**MF795868**	**MF795906**
*Neocucurbitaria rhamnicola*	KRx	*Rhamnus alaternus*	–	MF795781	MF795781	MF795823	MF795869	MF795907
*Neocucurbitaria rhamnioides*	C222	*Rhamnus saxatilis* subsp. *prunifolius*	–	MF795783	MF795783	MF795825	MF795871	MF795909
*Neocucurbitaria rhamnioides*	C223	*Rhamnus saxatilis* subsp*. prunifolius*	–	MF795784	MF795784	MF795826	MF795872	MF795910
***Neocucurbitaria rhamnioides***	**CBS 142395 = C118**	***Rhamnus myrtifolius***	**Holotype**	**MF795782**	**MF795782**	**MF795824**	**MF795870**	**MF795908**
***Neocucurbitaria ribicola***	**CBS 142394 = C55**	***Ribes rubrum***	**Holotype**	**MF795785**	**MF795785**	**MF795827**	**MF795873**	**MF795911**
*Neocucurbitaria ribicola*	C155	*Ribes rubrum*	–	MF795786	MF795786	MF795828	MF795874	MF795912
*Neocucurbitaria unguis-hominis*	CBS 111112	*Agapornis* sp.	–	LT623222	GQ387623	LT623279	–	LT623239
***Neocucurbitaria vachelliae***	**CBS 142397 = C192**	***Vachellia gummifera***	**Holotype**	**MF795787**	**MF795787**	**MF795829**	**MF795875**	**MF795913**
***Paracucurbitaria italica***	**CBS 234.92**	***Olea europaea***	**Holotype**	**LT623219**	**EU754176**	**LT623274**	**–**	**LT623235**
***Paracucurbitaria riggenbachii***	**CBS 248.79**	***Fraxinus excelsior*** **with bacterial canker**	**Holotype**	**LT903672**	**GQ387608**	**LT903673**	**–**	**LT900365**
***Parafenestella alpina***	**CBS 145263 = C198**	***Cotoneaster integerrimus***	**Holotype**	**MK356302**	**MK356302**	**MK357530**	**MK357574**	**MK357615**
*Parafenestella alpina*	C249	*Salix appendiculata*	–	MK356303	MK356303	MK357531	MK357575	MK357616
***Parafenestella austriaca***	**CBS 145262 = C152**	***Rosa canina***	**Holotype**	**MK356304**	**MK356304**	**MK357532**	**MK357576**	**MK357617**
***Parafenestella changchunensis***	**HMJAU 60182**	***Populus*** **L.**	**Holotype**	**OL996119**	**OL897170**	**–**	**OL944600**	**OL898719**
***Parafenestella faberi***	**MFLUCC 16-1451**	***Rosa canina***	**Holotype**	**KY563071**	**KY563074**	**–**	**–**	**–**
***Parafenestella germanica***	**CBS 145267 = C307**	***Corylus avellana***	**Holotype**	**MK356305**	**MK356305**	**MK357533**	**MK357577**	**MK357618**
*Parafenestella ostryae*	MFLU 16-0184	*Ostrya carpinifolia*	–	KY563072	KY563075	–	–	–
***Parafenestella pittospori***	**CPC 34462**	***Pittosporum tenuifolium***	**Holotype**	**MN562098**	**MN567606**	**–**	**–**	**–**
***Parafenestella pseudoplatani***	**CBS 142392 = C26**	***Acer pseudoplatanus***	**Holotype**	**MF795788**	**MF795788**	**MF795830**	**MF795876**	**MF795914**
***Parafenestella pseudosalicis***	**CBS 145264 = C301**	***Salix*** **cf. *alba***	**Holotype**	**MK356307**	**MK356307**	**MK357535**	**MK357579**	**MK357620**
***Parafenestella rosacearum***	**CBS 145268 = C309**	***Pyracantha coccinea***	**Holotype**	**MK356311**	**MK356311**	**MK357539**	**MK357583**	**MK357624**
*Parafenestella rosacearum*	C203	*Pyrus communis*	–	MK356308	MK356308	MK357536	MK357580	MK357621
*Parafenestella rosacearum*	C269	*Crataegus monogyna*	–	MK356309	MK356309	MK357537	MK357581	MK357622
*Parafenestella rosacearum*	C283	*Pyrus communis*	–	MK356310	MK356310	MK357538	MK357582	MK357623
*Parafenestella rosacearum*	C315	*Rosa canina*	–	MK356312	MK356312	MK357540	MK357584	MK357625
*Parafenestella rosacearum*	C320	*Sorbus aria*	–	MK356315	MK356315	MK357543	MK357587	–
*Parafenestella rosacearum*	CBS 145272 = FP11	*Prunus domestica*	–	MK356314	MK356314	MK357542	MK357586	MK357627
*Parafenestella rosacearum*	FM1	*Rosa canina*	–	MK356313	MK356313	MK357541	MK357585	MK357626
***Parafenestella salicis***	**CBS 145270 = C313**	***Salix alba***	**Neotype**	**MK356317**	**MK356317**	**MK357545**	**MK357589**	**MK357629**
*Parafenestella salicis*	C303	*Salix alba*	–	MK356316	MK356316	MK357544	MK357588	MK357628
***Parafenestella salicum***	**CBS 145269 = C311**	***Salix alba***	**Holotype**	**MK356318**	**MK356318**	**MK357546**	**MK357590**	**MK357630**
***Parafenestella tetratrupha***	**CBS 145266 = C304**	***Alnus glutinosa***	**Epitype**	**MK356319**	**MK356319**	**MK357547**	**MK357591**	**MK357631**
***Parafenestella ulmi***	**HMJAU 60178**	***Ulmus pumila*** **L.**	**Holotype**	**OL996115**	**OL897166**	**OL944501**	**OL944596**	**OL898723**
***Parafenestella ulmi***	**HMJAU 60179**	***Ulmus pumila*** **L.**	**Isotype**	**OL996116**	**OL897167**	**OL944502**	**OL944597**	**OL898717**
***Parafenestella ulmicola***	**HMJAU 60180**	***Ulmus pumila*** **L.**	**Holotype**	**OL996117**	**OL897168**	**OL944503**	**OL944598**	**OL898724**
***Parafenestella ulmicola***	**HMJAU 60181**	***Ulmus pumila*** **L.**	**Isotype**	**OL996118**	**OL897169**	**OL944504**	**OL944599**	**OL898718**
***Parafenestella vindobonensis***	**CBS 145265 = C302**	***Salix babylonica***	**Holotype**	**MK356320**	**MK356320**	**MK357548**	**MK357592**	**MK357632**
***Protofenestella ulmi***	**CBS 143000 = FP5**	***Ulmus minor***	**Holotype**	**MF795791**	**MF795791**	**MF795833**	**MF795879**	**MF795915**
***Pyrenochaeta nobilis***	**CBS 407.76 = AFTOL-ID 1856**	***Laurus nobilis leaves***	**Neotype**	**MF795792**	**MF795792**	**MF795834**	**MF795880**	**MF795916**
***Pyrenochaetopsis americana***	**UTHSC DI16-225**	**–**	**Holotype**	**LT592912**	**LN907368**	**LT593050**	**–**	**LT592981**
***Pyrenochaetopsis confluens***	**CBS 142459**	**Deep tissue/ fluids from human blood sample**	**Holotype**	**LT592950**	**LN907446**	**LT593089**	**–**	**LT593019**
***Seltsamia galinsogisoli***	**CBS 140956 = CGMCC 3.17981 =SYPF 7336**	**Soil of a *Galinsoga parviflora***	**Epitype**	**KU759584**	**KU759581**	**–**	**–**	**–**
*Seltsamia* sp.	EAB-67-11b	Emerald ash borer	–	MT777389	–	–	–	–
*Seltsamia* sp.	SGSF207	–	–	MK192899	–	–	–	–
***Seltsamia ulmi***	**CBS 143002 = L150**	***Ulmus glabra***	**Holotype**	**MF795794**	**MF795794**	**MF795836**	**MF795882**	**MF795918**
***Synfenestella pyri***	**CBS 144855 = C297**	***Pyrus communis***	**Holotype**	**MK356321**	**MK356321**	**MK357549**	**MK357593**	**MK357633**
*Synfenestella sorbi*	C298	*Sorbus aucuparia*	–	MK356325	MK356325	MK357553	MK357597	MK357636
***Synfenestella sorbi***	**CBS 144858 = C196**	***Sorbus aucuparia***	**Holotyp** **e**	**MK356324**	**MK356324**	**MK357552**	**MK357596**	**MK357635**
***Synfenestella sorbi***	**CBS 144862 = FR**	***Sorbus aucuparia***	**Epitype**	**MK356322**	**MK356322**	**MK357550**	**MK357594**	**MK357634**
*Synfenestella sorbi*	FRa	*Sorbus aucuparia*	–	MK356323	MK356323	MK357551	MK357595	–

**Table 3:** Synopsis of sexual morph characteristics of eleven *Parafenestella* species with the newly introduced species in bold.

Taxon	Sexual Morph		
	Ascomata	Asci	Ascospores
*P. alpina*	240–375 μm diam, globose, subglobose or pyriform, usually tightly aggregated in bark on a perithecial host fungus in small numbers, with brown to black, subicular hyphae.	170–208 × 18.5–21.5 μm, cylindrical to oblong, a short stipe and simple or knob-like base, containing 6–8 ascospores in uniseriate arrangement.	24–30.5 × 12–14 μm, typically ellipsoid to fusoid often inequilateral, pale or yellowish-brown, eventually dark brown, with 7–15 transverse and 2–4 longitudinal septa.
*P. austriaca*	283–431 μm diam, subglobose to pyriform, scattered or aggregated, basally and laterally surrounded by subhyaline to dark brown subicular hyphae.	159–205 × 16–19.5 μm, cylindrical, with a short stipe and simple or knob-like base, containing 4–8 ascospores in uniseriate arrangement.	27–32.5 × 13–15 µm, broadly ellipsoid, symmetric, dark brown or dark reddish-brown, with 9–14 distantly spaced transverse and 3–5 longitudinal septa.
*P. changchunensis*	280 × 353 μm, globose to depressed globose, solitary or aggregated forming visible black bumps submerged under bark.	95–138 × 16–21 μm, broad cylindrical, short-pedicellate, curved, some curved, 6–8 spores ocular chamber is not visible at maturity, uniseriate arrangement.	18–25 × 8–13 μm, fusiform to oval, light yellow to dark brown, developing 2 main septa, 4–6 transverse septa, 2–3 longitudinal septa.
*P. faberi*	300–500 μm diam, tightly or loosely aggregated in small numbers, with ostiolar, partly erumpent through bark fissures, maxing with *Cytospora* species.	135–180 × 18.5–23.5 μm, cylindrical to oblong or narrowly clavate, a short stipe and simple or knob-like base, 4–8 ascospores in uniseriate to partly biseriate arrangement.	28.5–36 × 12.5–16 µm, variable in shape, pale or yellowish-brown to dark brown, with 1–4 main septa, 7–14 transverse and 1–5 longitudinal septa.
*P. germanica*	230–450 μm diam, black, solitary or in small groups on inner bark or on the ostiolar level of old *Diaporthe decedens.*	140–173 × 17.5–22 μm, cylindrical to oblong, with a short stipe and simple or knob-like base, containing 2–8 ascospores (obliquely or overlapping), uniseriate arrangement.	29–39.5 × 13–16.5 μm, ellipsoid to broadly fusoid, turning yellow to yellow-brown to dark brown, with 1–3 main septa, 8–15 transverse and 3–6 longitudinal septa.
*P. parasalicum*	270–400 μm diam, immersed in bark, globose, subglobose or pyriform, forming groups, maxing with *Cytospora* species.	185–219 × 22–27 μm, cylindrical to oblong, with a short stipe and simple or knob-like base, containing 4–8 ascospores (overlapping, obliquely), uniseriate to partly biseriate arrangement.	36–44 × 15.8–19.3 μm, fusoid or ellipsoid, yellow-brown to dark brown, with 2 main septa, 11–16 distinct transverse septa and 3–5 longitudinal septa.
*P. pseudosalicis*	300–400 diam, subglobose to subpyriform, immersed in bark or on ascomata of an effete perithecial fungus, often with concave apex, covered with subicular hyphae.	186–215 × 17.5–19 μm, cylindrical to oblong, with a short stipe and simple or knob-like base, containing 4–8 ascospores in uniseriate arrangement.	25–29 × 12–14 μm, ellipsoid, yellow-brown to dark brown, with 1–3 main septa, 7–11 transverse and 2–4 longitudinal septa, with minute guttules.
*P. rosacearum*	285–432 μm diam, globose, subglobose to subpyriform, immersed on often blackened inner bark, scattered or in small groups, erumpent through bark fissures.	181–240 × 19–22 μm, cylindrical to oblong, with a short-contorted stipe and simple or knob-like base, containing 2–8 ascospores in uniseriate, rarely partly biseriate arrangement.	28–35 × 13.5–16.5 μm, ellipsoid, symmetric to inequilateral, yellow-brown to dark brown, with 1–3 main septa, 7–15 transverse and 2–5 longitudinal septa.
*P. salicis*	275–442 μm diam, globose, subglobose to pyriform or subconical, immersed below the epidermis on inner bark, partly erumpent through bark fissures.	141–188 × 16–19 μm, cylindrical to oblong, with a short stipe and simple or knob-like base, containing 1–8 ascospores in (obliquely) uniseriate to partly biseriate arrangement.	23–29 × 11–13.5 μm, ellipsoid to fusoid, symmetric, golden yellow-brown (when fresh) to dark brown, with 1–3 main septa, 5–11 transverse and 1–3 longitudinal septa.
*P. salicum*	270–420 diam, globose, subglobose or pyriform, immersed in bark, the inner bark layers connected to the host, scattered or aggregate, cover with subicular hyphae.	181–228 × 19.5–24 μm, cylindrical, with a short stipe and simple or knob-like base, containing 6–8 ascospores in (overlapping) uniseriate arrangement.	27–33 × 12.5–16 μm, broadly ellipsoid to broadly fusoid, first 2-celled and hyaline, turning golden yellow to dark brown or dark reddish-brown, with 9 –14 transverse and 3–4 longitudinal septa.
*P. tetratrupha*	300–500 μm diam, globose, subglobose or pyriform, immersed, tightly or loosely aggregated in whitish to dark brown subiculum, erumpent through fissures.	154–229 × 18.5–22.2 μm, cylindrical to oblong, with a short stipe and simple or knob-like base, containing 2–8 ascospores in uniseriate arrangement.	26.5–33.5 × 13–16.5 μm, ellipsoid, yellow-brown to reddish-brown to dark brown, with 1–3 main septa, 8–17 distinct transverse and 2–4 longitudinal septa.
*P. ulmi*	170–225 × 194–260 μm, globose to ellipsoid, immersed under the host epidermis, visible as black spots or having a convex surface.	115–181 × 11–15 μm, cylindrical, mostly curved, short-pedicellate, containing 8 ascospores, uniseriate to partially overlapping.	18–24 × 8–12 μm, broadly ellipsoid, yellowish to brown, with 5–8 transversely septate, 1–2 longitudinal septa.
*P. ulmicola*	242–434 × 310–462 μm, globose to subglobose, on the surface, semi-immersed, visible as a convex hemisphere, with a papilla.	105–153 × 11–14 μm, broad cylindrical, some curved, short-pedicellate, containing 8 ascospores, short-pedicellate, uniseriate, rarely overlapping.	17–22 × 8–12 μm, broadly oval, yellowish to brown, with 4–8 transversely septate and 1–3 vertical septate.
*P. vindobonensis*	308–425 μm diam, globose, subglobose or pyriform, immersed in bark, partially erumpent, tightly aggregated in small groups on inner bark mixing with pseudostromata of a *Cytospora* sp.	179–214 × 13.5–15.5 μm, cylindrical, with a short stipe and simple or knob-like base, containing 4–8 ascospores in uniseriate arrangement.	24.5–30.5 × 9.5–11 μm, oblong, fusoid or narrowly ellipsoid, turning yellowish to medium brown, 1–6 main septa, when mature with 7–11 thick transverse and 1–3 septa, containing minute droplets.

## Data Availability

All sequences generated in this study were submitted to GenBank.

## Supplementary Materials

The following are available online at https://www.mdpi.com/article/10.3390/jof8090905/s1, Figure S1: The best-scoring RAxML tree based on a concatenated ITS dataset. Figure S2: The best-scoring RAxML tree based on a concatenated LSU dataset. Figure S3: The best-scoring RAxML tree based on a concatenated *rpb*2 dataset. Figure S4: The best-scoring RAxML tree based on a concatenated *tef*1*-α* dataset. Figure S5: The best-scoring RAxML tree based on a concatenated *tub*2 dataset. Figure S6. The best-scoring RAxML tree based on a concatenated ITS, LSU, *rpb*2, *tef*1*-α* and *tub*2 dataset. Figure S7. Phylogram generated from maximum parsimony analysis based on combined ITS, LSU, *rpb*2, *tef*1*-α* and *tub*2 dataset. Figure S8: Phylogram generated from ASAP analysis using LSU sequence data. Figure S9: Phylogram generated from ASAP analysis using *rpb*2 sequence data. Figure S10: Phylogram generated from ASAP analysis using *tef*1*-α* sequence data. Figure S11: Phylogram generated from ASAP analysis using ITS, LSU, *rpb*2, *tef*1*-α* and *tub*2 dataset. Figure S12: The best-scoring RAxML tree based on a concatenated ITS + *rpb*2 dataset. Figure S13: The best-scoring RAxML tree based on a concatenated ITS + *tef*1*-α* dataset. Figure S14: The best-scoring RAxML tree based on a concatenated ITS + *tub*2 dataset.
