# Supplementary material for: Diversity of Ascomycota in Jilin: Introducing Novel Woody Litter Taxa in Cucurbitariaceae
Source: J Fungi (Basel). 2022 Aug 26;8(9):905. doi: 10.3390/jof8090905 (PMC9501381; doi:10.3390/jof8090905)
Supplement: Supplementary file 1 [file jof-08-00905-s001.zip › jof-1842178-supplementary.pdf]

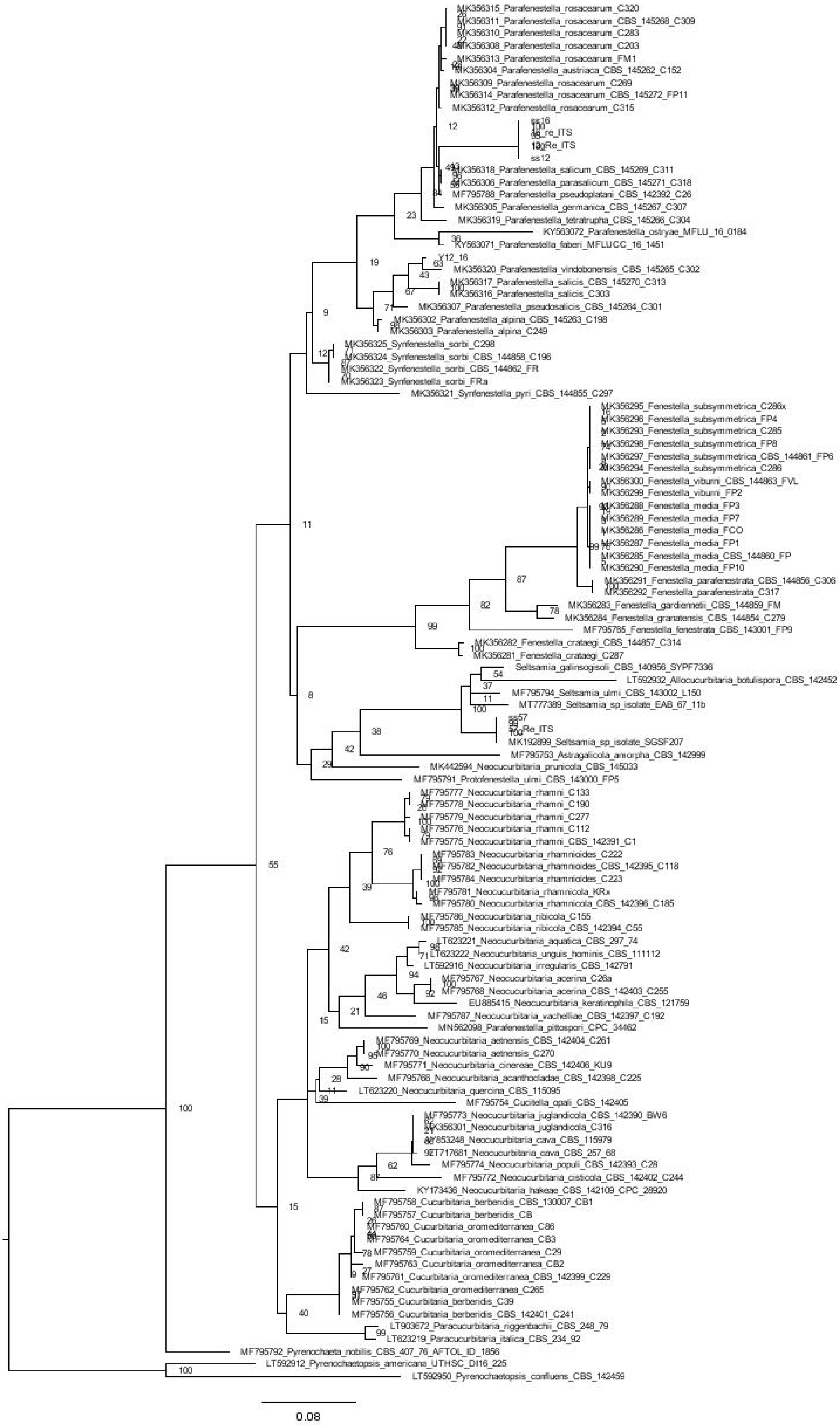

Figure S1. The best-scoring RAxML tree based on a concatenated ITS dataset.

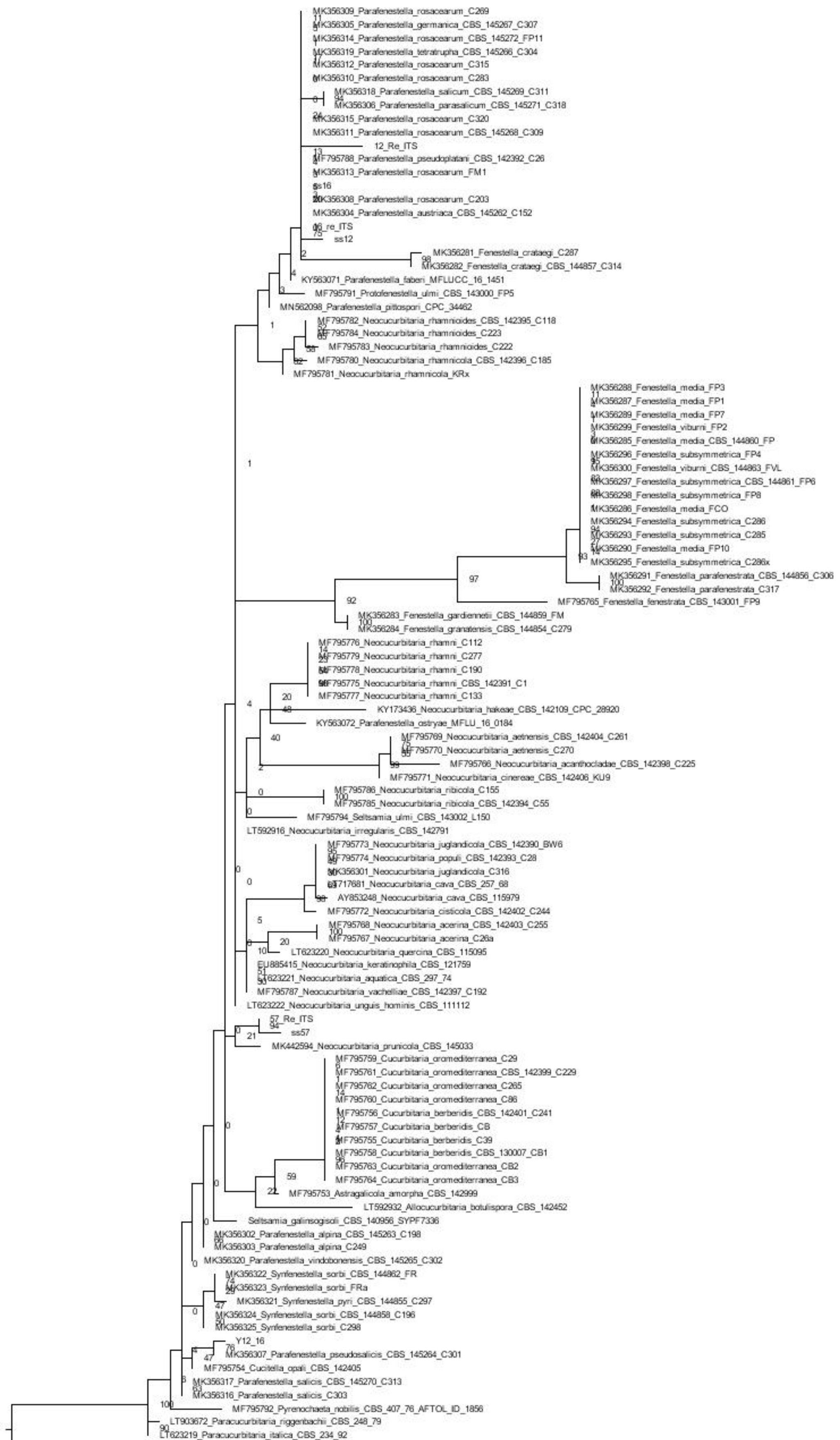

Figure S2. The best-scoring RAxML tree based on a concatenated LSU dataset.

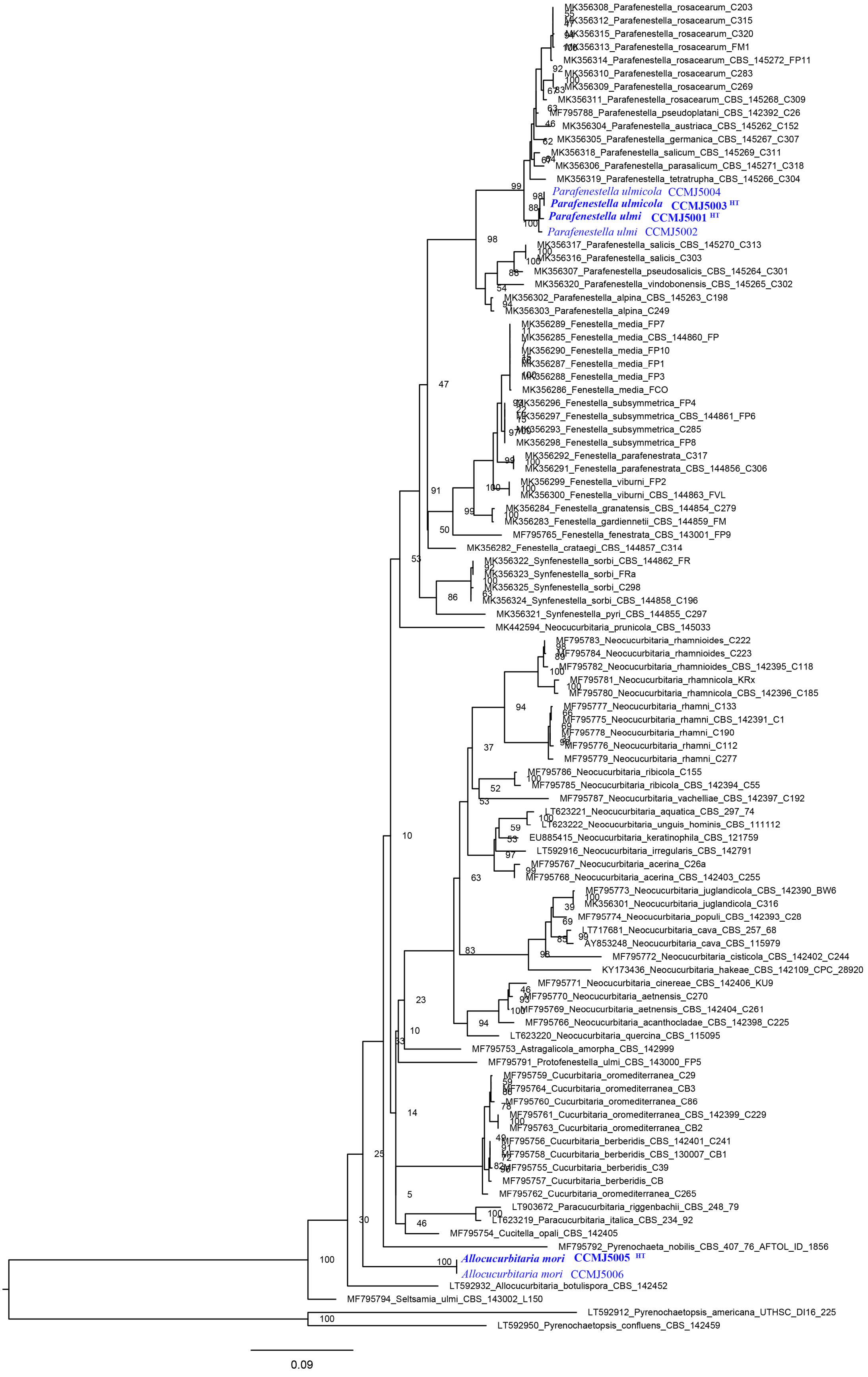

Figure S3. The best-scoring RAxML tree based on a concatenated *rpb*2 dataset.

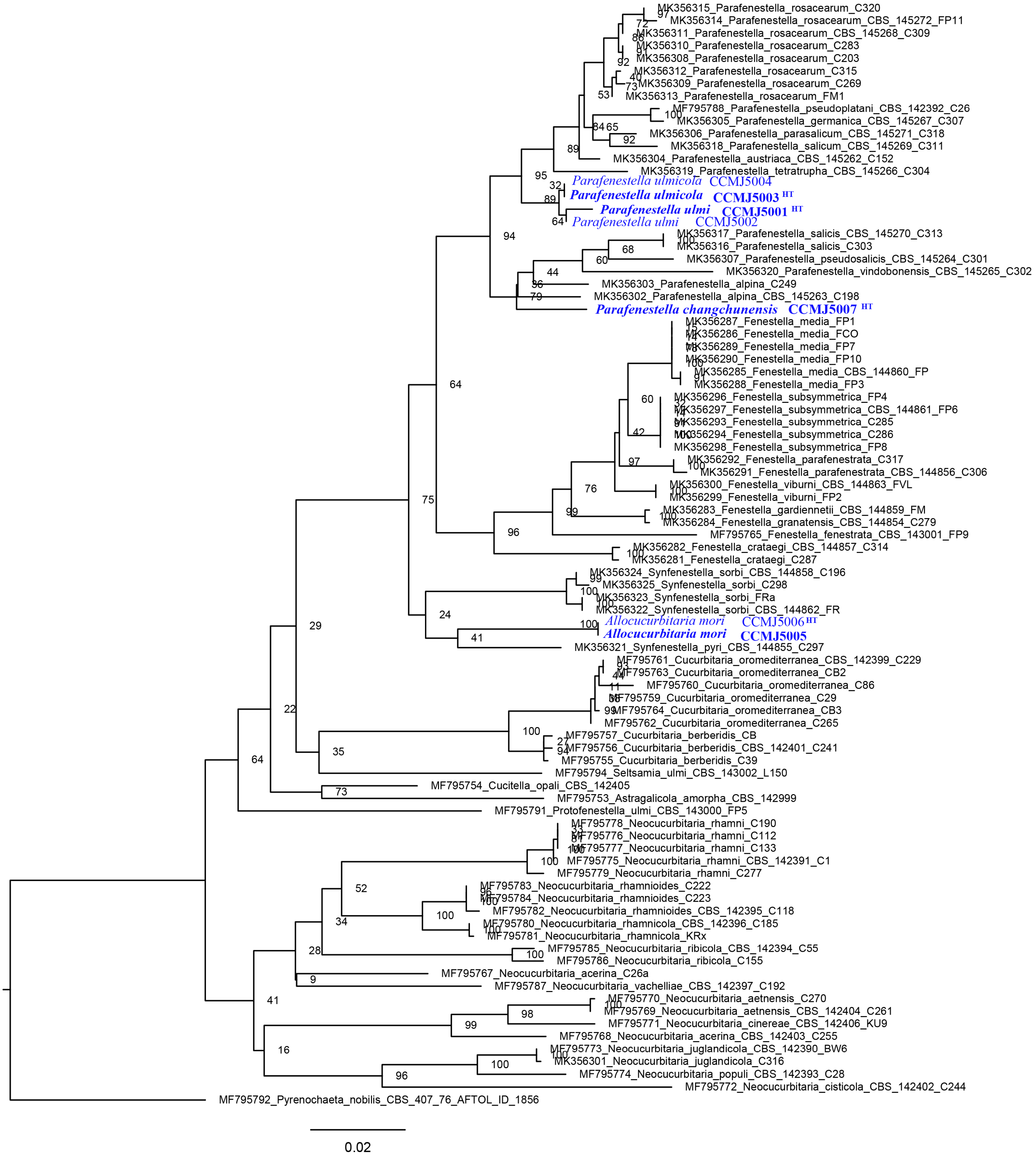

Figure S4. The best-scoring RAxML tree based on a concatenated *tef*1-*α* dataset.

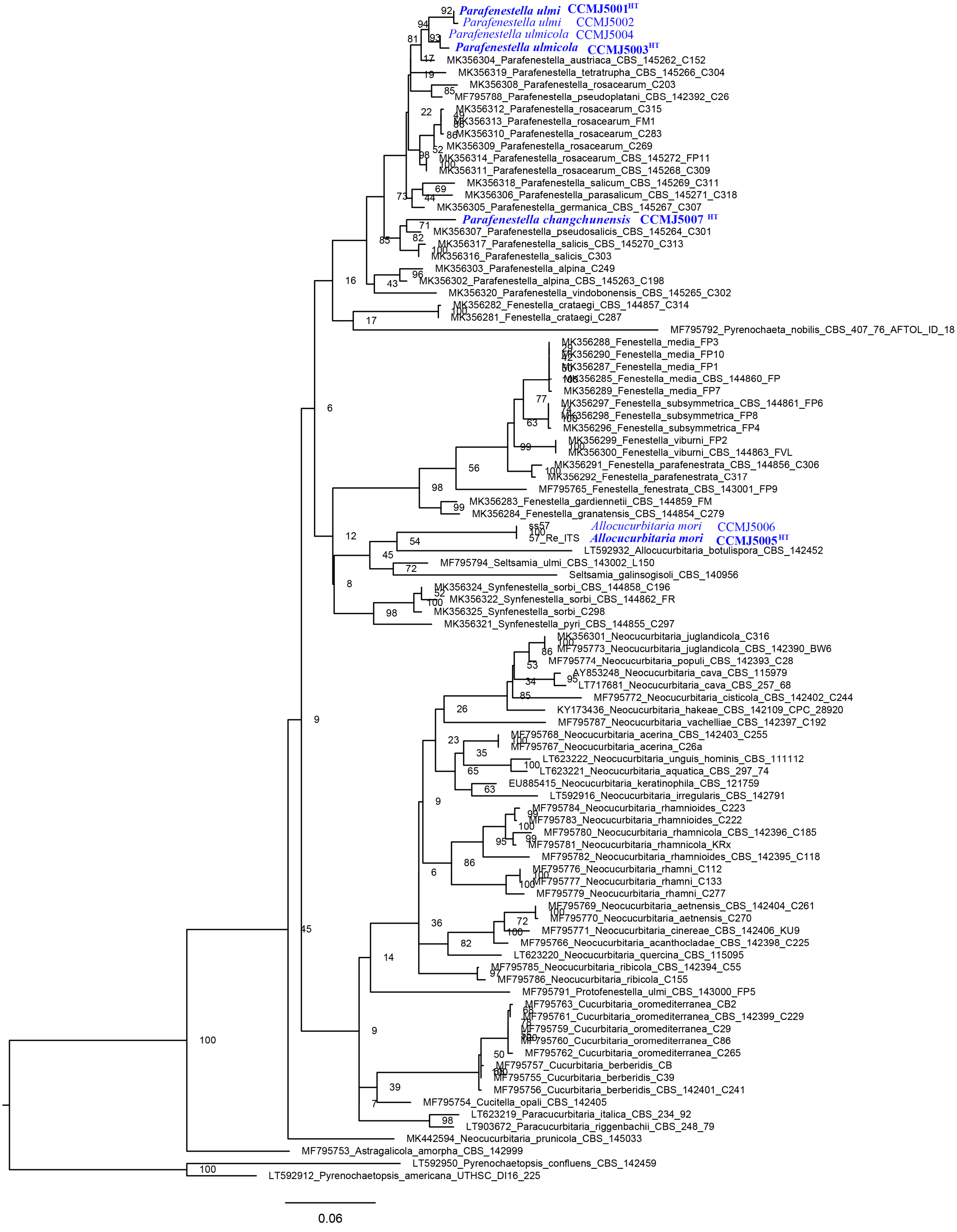

Figure S5. The best-scoring RAxML tree based on a concatenated *tub*2 dataset.

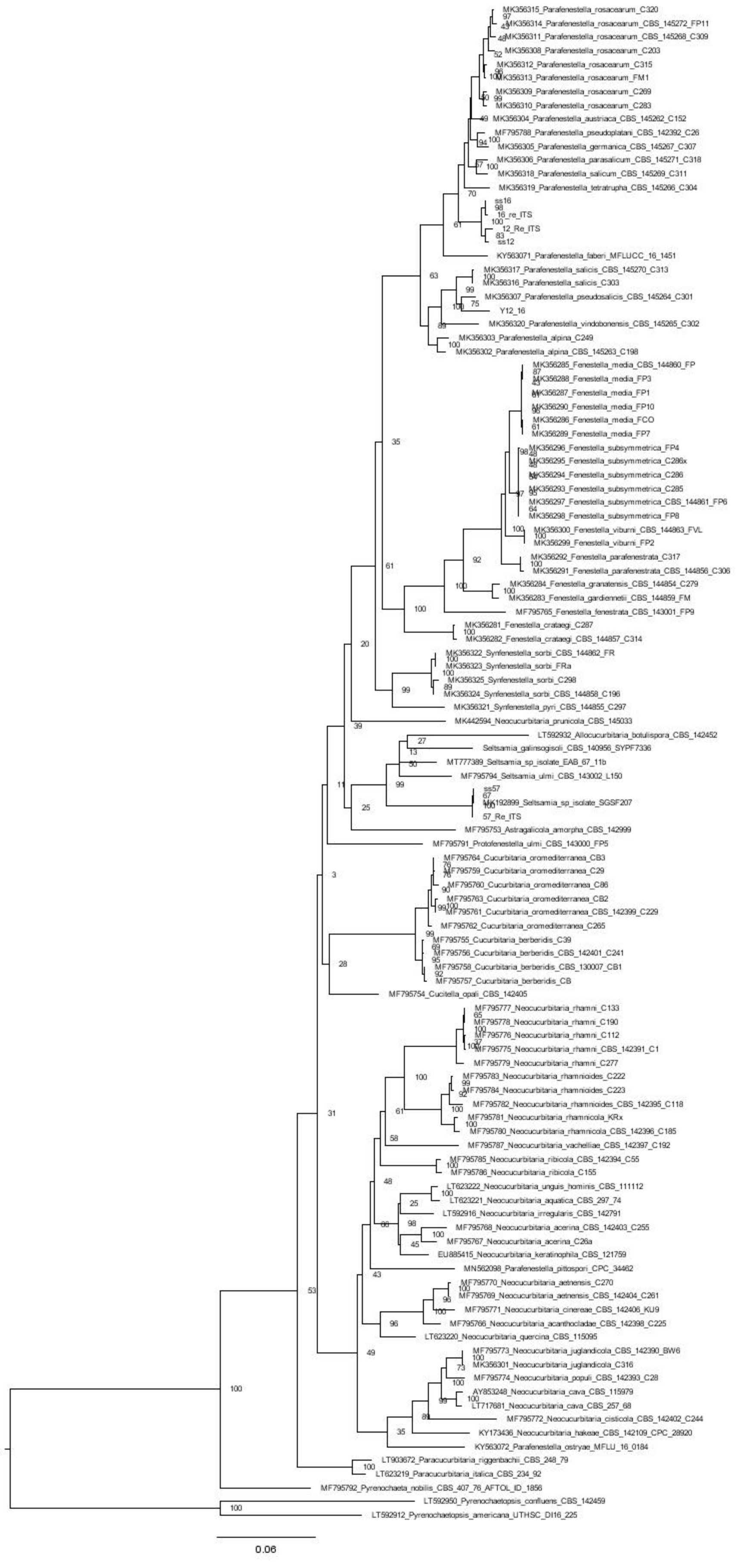

Figure S6. The best-scoring RAxML tree based on a concatenated ITS, LSU, *rpb*2, *tef*1-*α* and *tub*2 dataset.

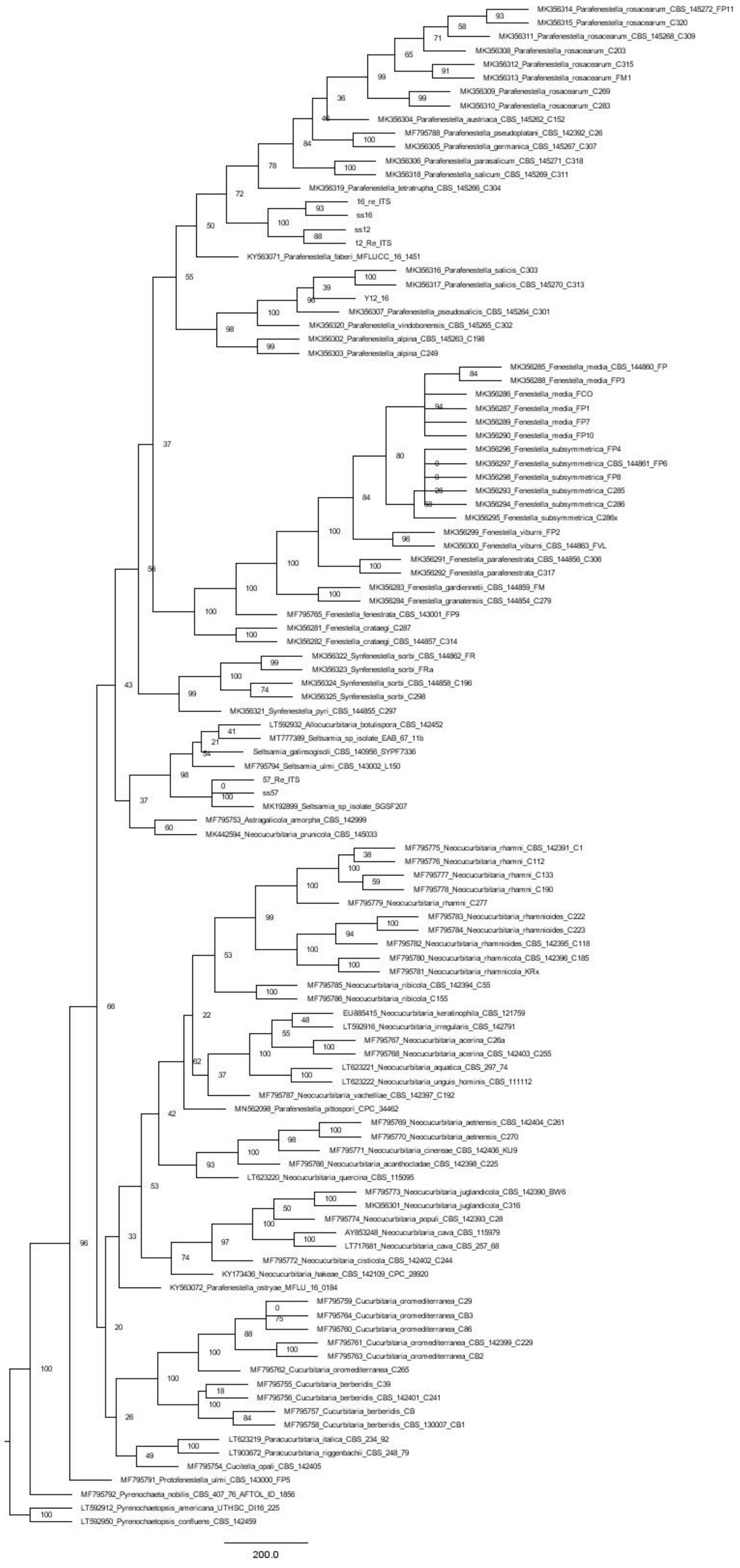

Figure S7. Phylogram generated from Maximum parsimony analysis based on combined ITS, LSU, *rpb*2, *tef*1-α and *tub*2 dataset.

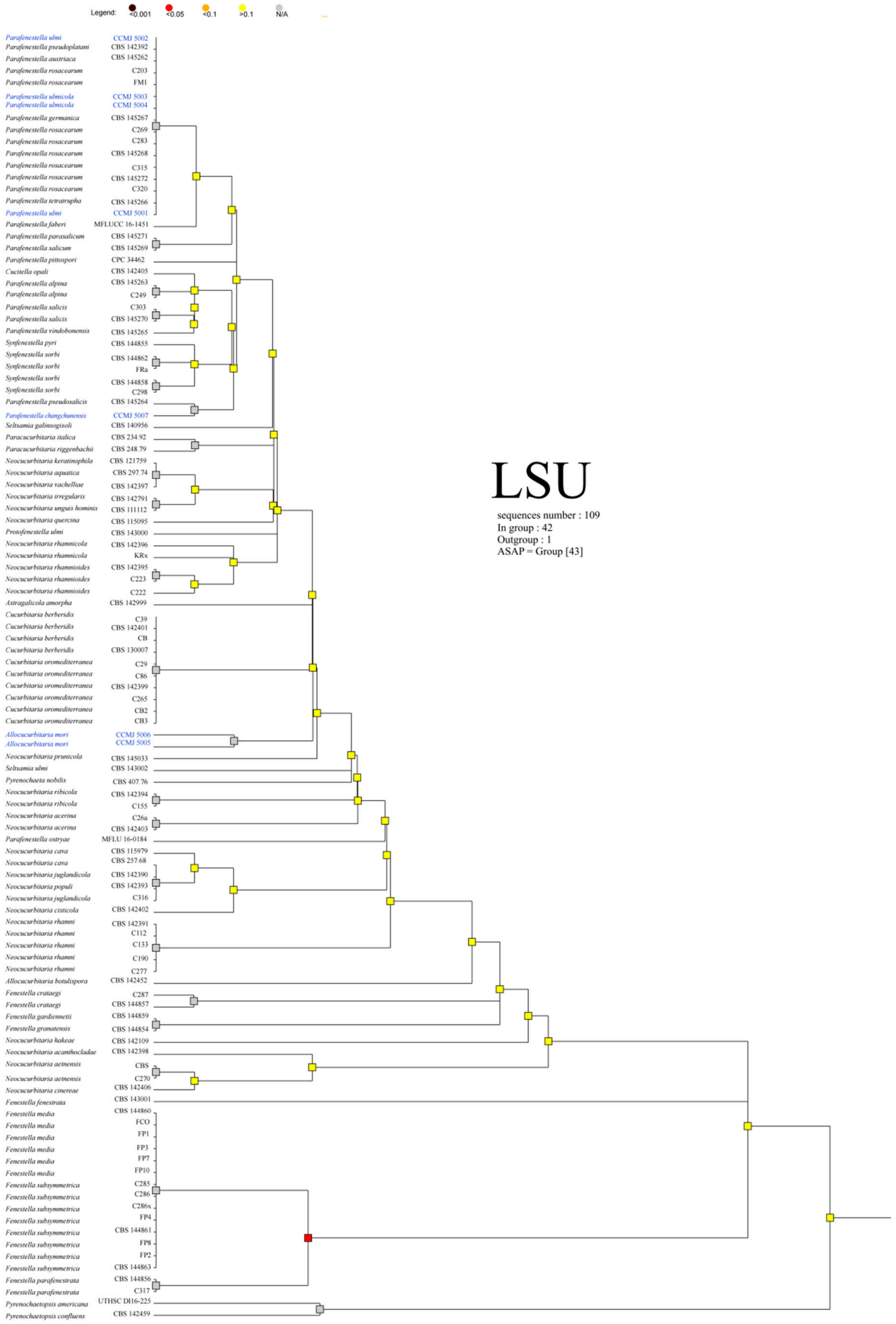

Figure S8. Phylogram generated from ASAP analysis using LSU sequence data.

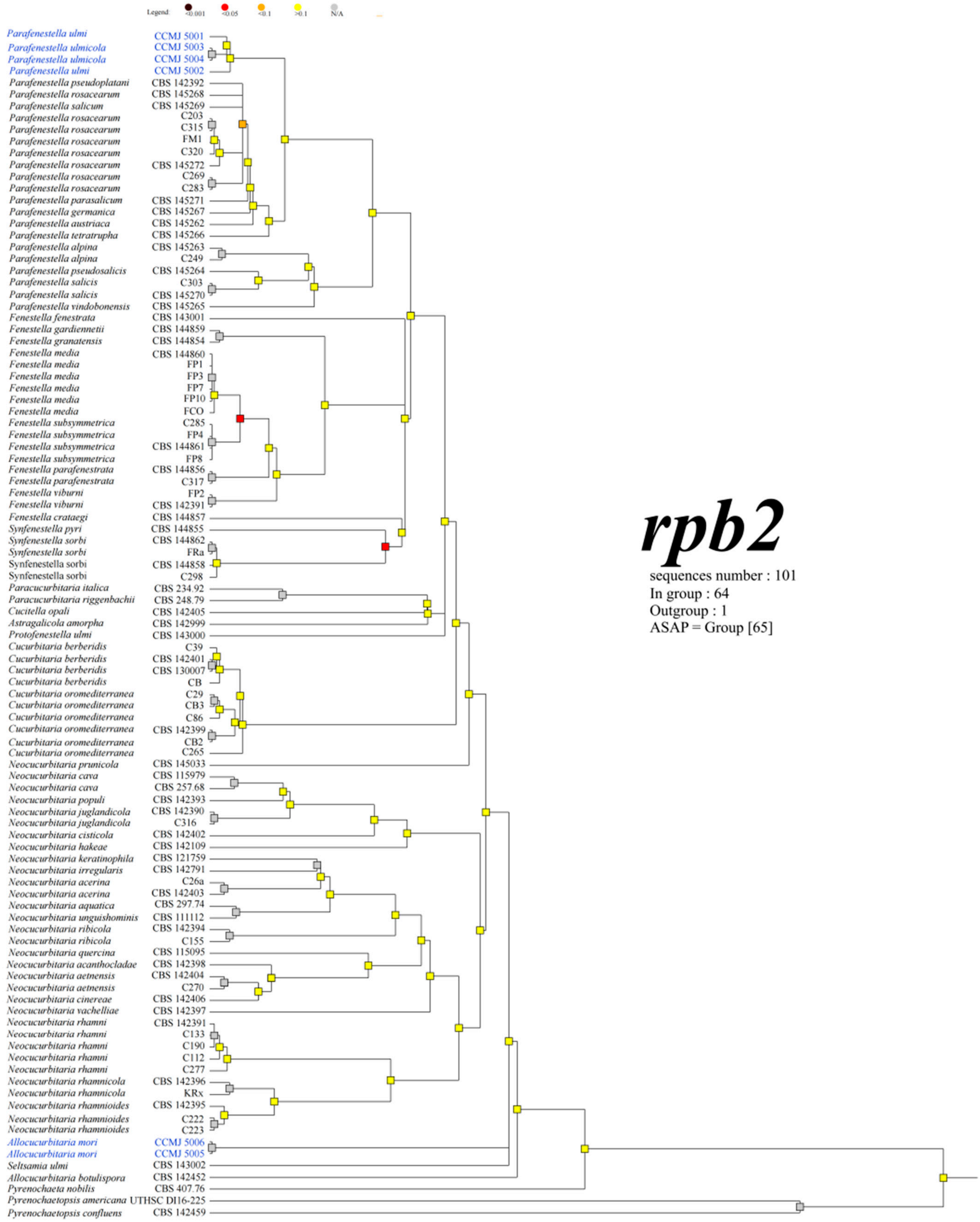

Figure S9. Phylogram generated from ASAP analysis using *rpb*2 sequence data.

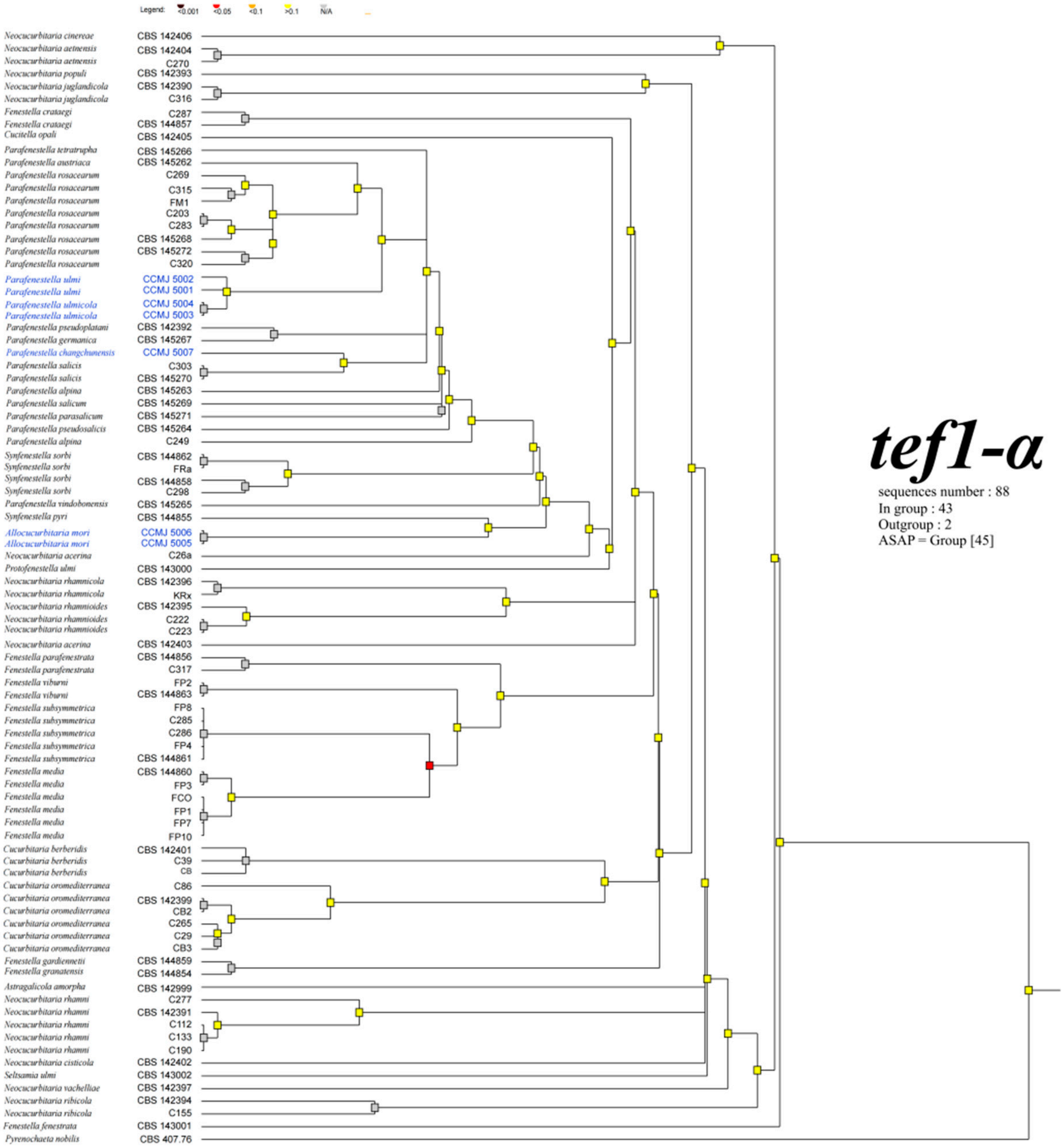

Figure S10. Phylogram generated from ASAP analysis using *tef*1-*α* sequence data.

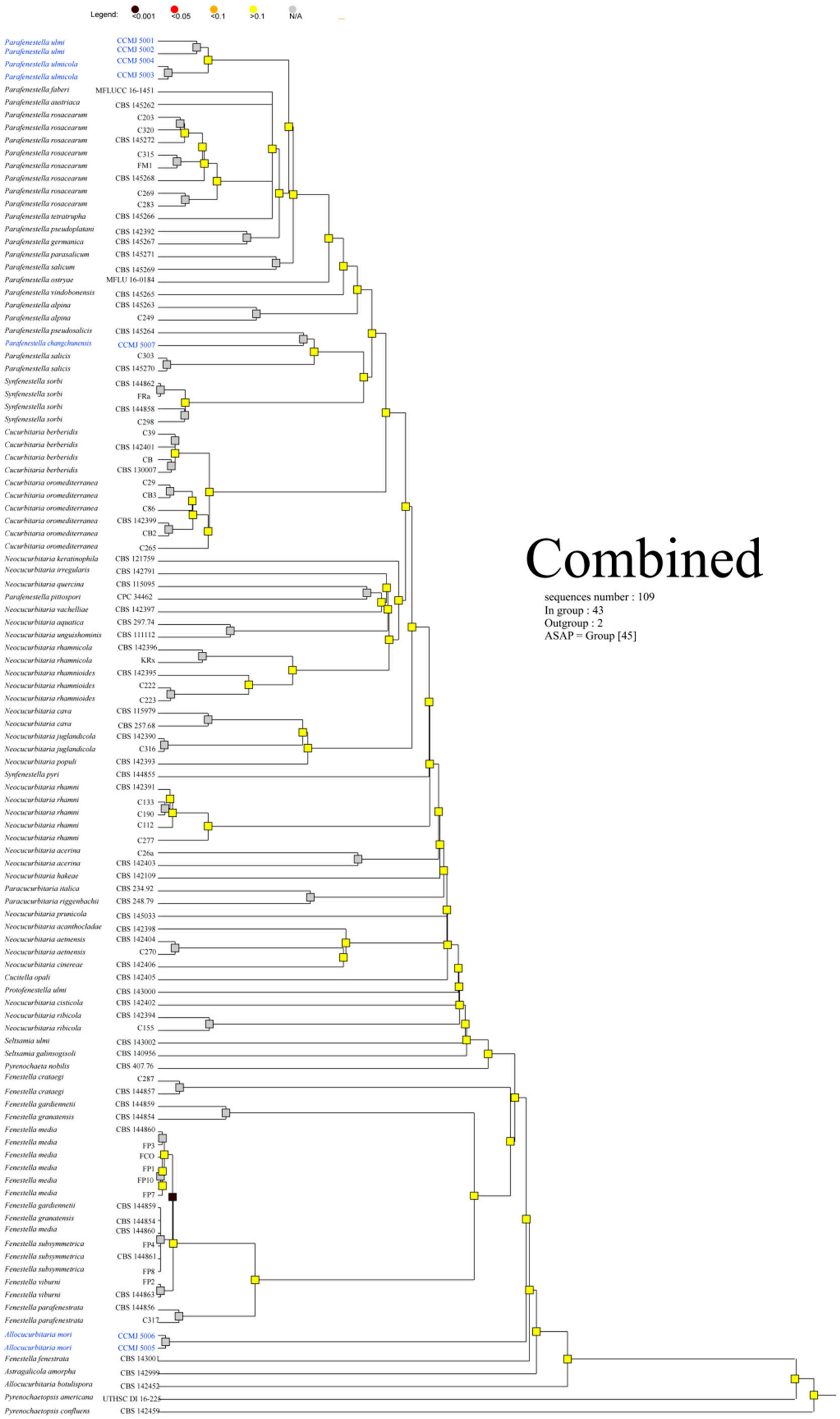

Figure S11. Phylogram generated from ASAP analysis using ITS, LSU, *rpb*2, *tef*1-*α* and *tub*2 dataset.

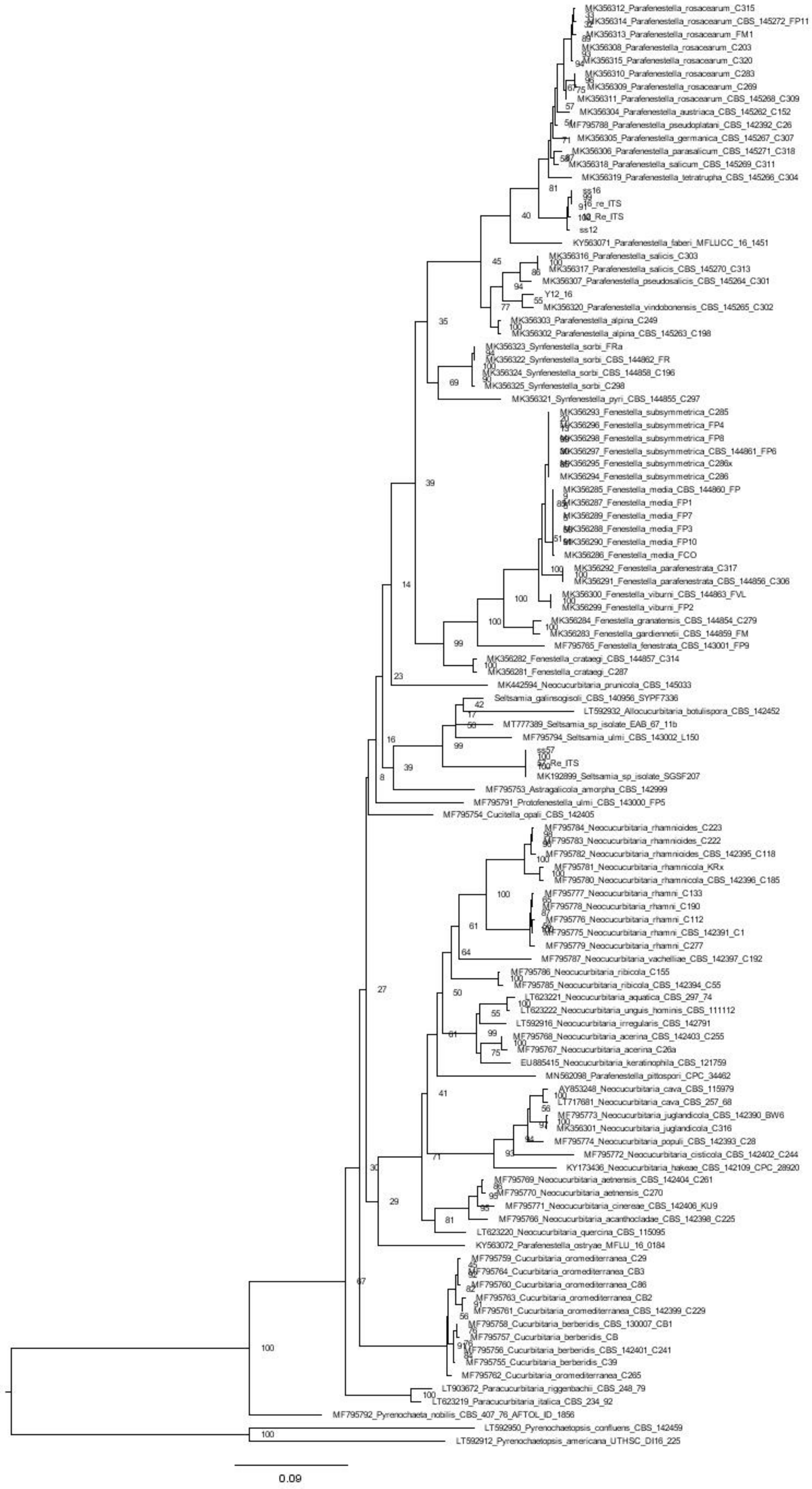

Figure S12. The best-scoring RAxML tree based on a concatenated ITS+*rpb*2 dataset.

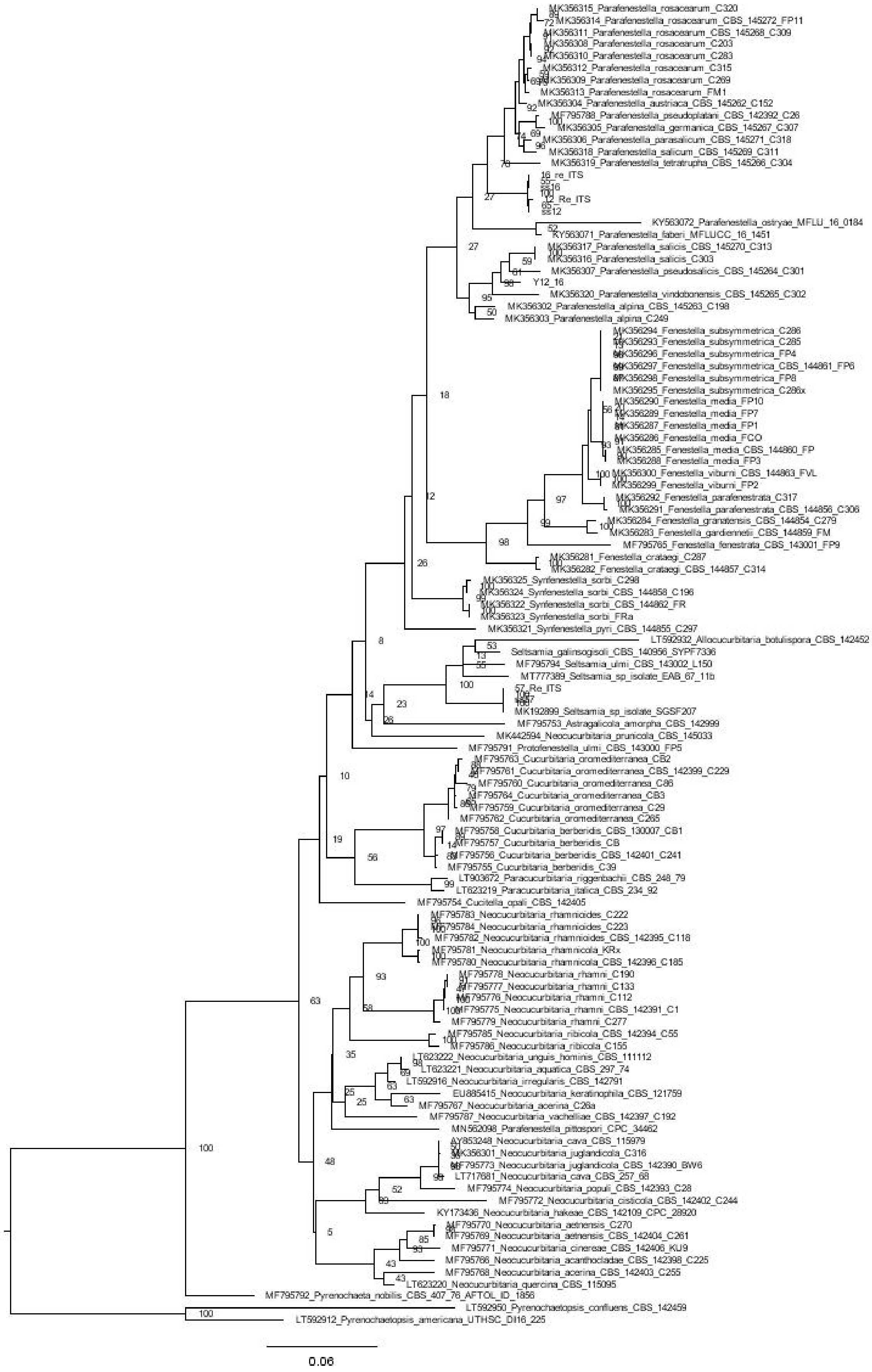

Figure S13. The best-scoring RAxML tree based on a concatenated ITS+*tef*1-α dataset.

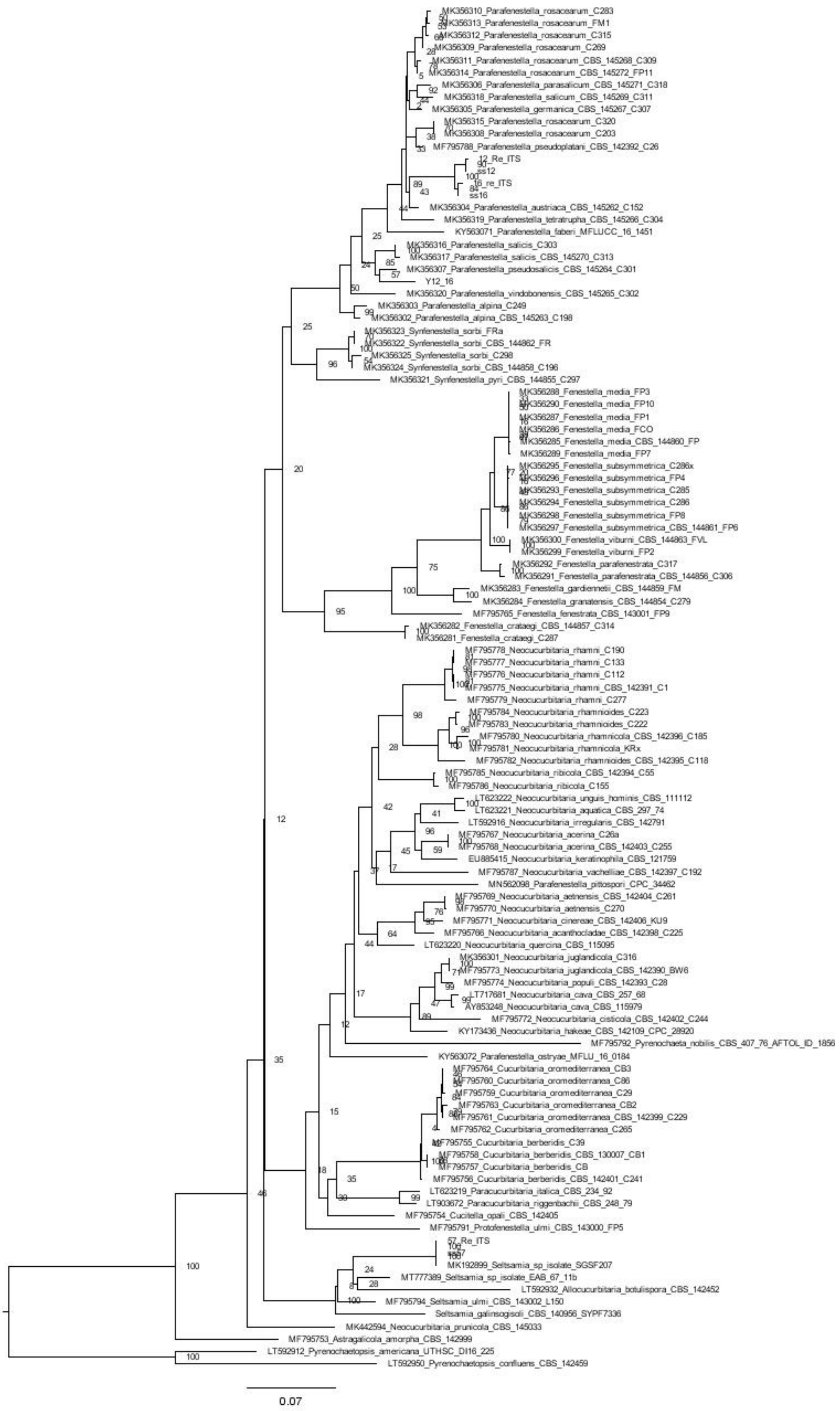

Figure S14. The best-scoring RAxML tree based on a concatenated ITS+*tub*2 dataset.
